# Isoamphipathic antibacterial molecules regulating activity and toxicity through positional isomerism†

**DOI:** 10.1039/d2sc06065e

**Published:** 2023-04-06

**Authors:** Swagatam Barman, Sudip Mukherjee, Logia Jolly, Cassandra Troiano, Alessandro Grottesi, Debajyoti Basak, Paolo Calligari, Brinta Bhattacharjee, Gianfranco Bocchinfuso, Lorenzo Stella, Jayanta Haldar

**Affiliations:** a Antibacterial Research Laboratory, New Chemistry Unit, Jawaharlal Nehru Centre for Advanced Scientific Research (JNCASR) Jakkur Bangalore 560064 India jayanta@jncasr.ac.in; b Department of Chemical Science and Technologies, University of Rome Tor Vergata via della Ricerca Scientifica, 1 00133 Rome Italy; c Cineca Via dei Tizii, 6 00185 Rome Italy; d School of Advanced Materials, Jawaharlal Nehru Centre for Advanced Scientific Research (JNCASR) Jakkur Bangalore 560064 India

## Abstract

Peptidomimetic antimicrobials exhibit a selective interaction with bacterial cells over mammalian cells once they have achieved an optimum amphiphilic balance (hydrophobicity/hydrophilicity) in the molecular architecture. To date, hydrophobicity and cationic charge have been considered the crucial parameters to attain such amphiphilic balance. However, optimization of these properties is not enough to circumvent unwanted toxicity towards mammalian cells. Hence, herein, we report new isoamphipathic antibacterial molecules (IAMs: **1–3**) where positional isomerism was introduced as one of the guiding factors for molecular design. This class of molecules displayed good (MIC = 1–8 μg mL^−1^ or μM) to moderate [MIC = 32–64 μg mL^−1^ (32.2–64.4 μM)] antibacterial activity against multiple Gram-positive and Gram-negative bacteria. Positional isomerism showed a strong influence on regulating antibacterial activity and toxicity for *ortho* [IAM-1: MIC = 1–32 μg mL^−1^ (1–32.2 μM), HC_50_ = 650 μg mL^−1^ (654.6 μM)], *meta* [IAM-2: MIC = 1–16 μg mL^−1^ (1–16.1 μM), HC_50_ = 98 μg mL^−1^ (98.7 μM)] and *para* [IAM-3: MIC = 1–16 μg mL^−1^ (1–16.1 μM), HC_50_ = 160 μg mL^−1^ (161.1 μM)] isomers. Co-culture studies and investigation of membrane dynamics indicated that *ortho* isomer, IAM-1 exerted more selective activity towards bacterial over mammalian membranes, compared to *meta* and *para* isomers. Furthermore, the mechanism of action of the lead molecule (IAM-1) has been characterized through detailed molecular dynamics simulations. In addition, the lead molecule displayed substantial efficacy against dormant bacteria and mature biofilms, unlike conventional antibiotics. Importantly, IAM-1 exhibited moderate *in vivo* activity against MRSA wound infection in a murine model with no detectable dermal toxicity. Altogether, the report explored the design and development of isoamphipathic antibacterial molecules to establish the role of positional isomerism in achieving selective and potential antibacterial agents.

## Abstract 2

Peptidomimetic antimicrobials exhibit a selective interaction with bacterial cells over mammalian cells once they have achieved an optimum amphiphilic balance (hydrophobicity/hydrophilicity) in the molecular architecture.

## Graphical abstract

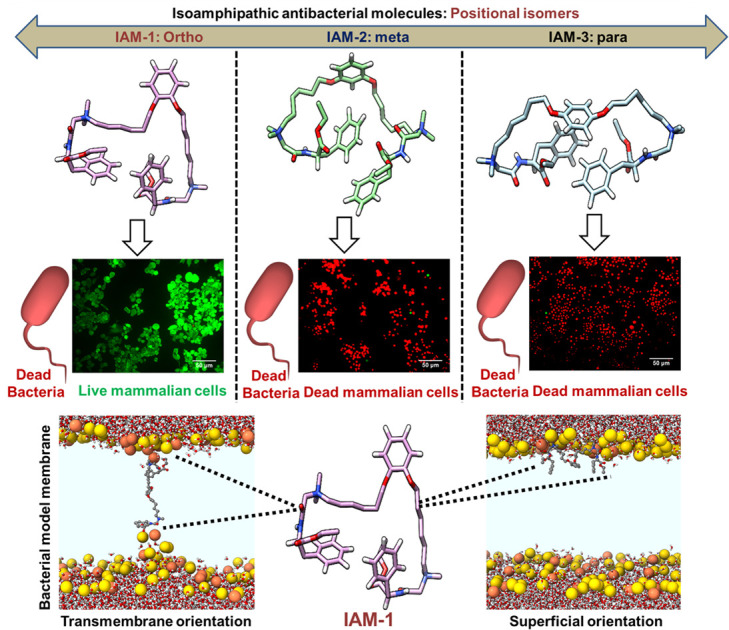

## Introduction

The rapid emergence of antimicrobial resistance (AMR) and chronic and recurrent infections owing to bacterial biofilm formation and predominance of dormant bacterial sub-population are accountable for huge global mortality.^1–9^ Another concern is a declining arsenal in antibiotic pipelines.^10,11^ Hence, various organizations, including the World Health Organization, raised the alarm for the urgent development of a new class of antimicrobial agents.^12^ Towards this aim, bacterial membrane targeting peptidomimetic antimicrobials have received significant attention, particularly because of the slower development of bacterial resistance against them.^13–26^ Nevertheless, these peptidomimetic molecules often suffer from unwanted toxicity toward mammalian cells. Various research groups, including ours, observed that in general, an increment in hydrophobicity in any peptidomimetic molecular design is associated with toxic effects.^13–16,18–27,40^ On the other side, the reduction in overall hydrophobicity produces less toxic molecules with compromised antibacterial efficacy. Thus, studying a new parameter for the fine structural tuning of peptidomimetic antibacterial molecules has significant relevance.

**Fig. 1:**
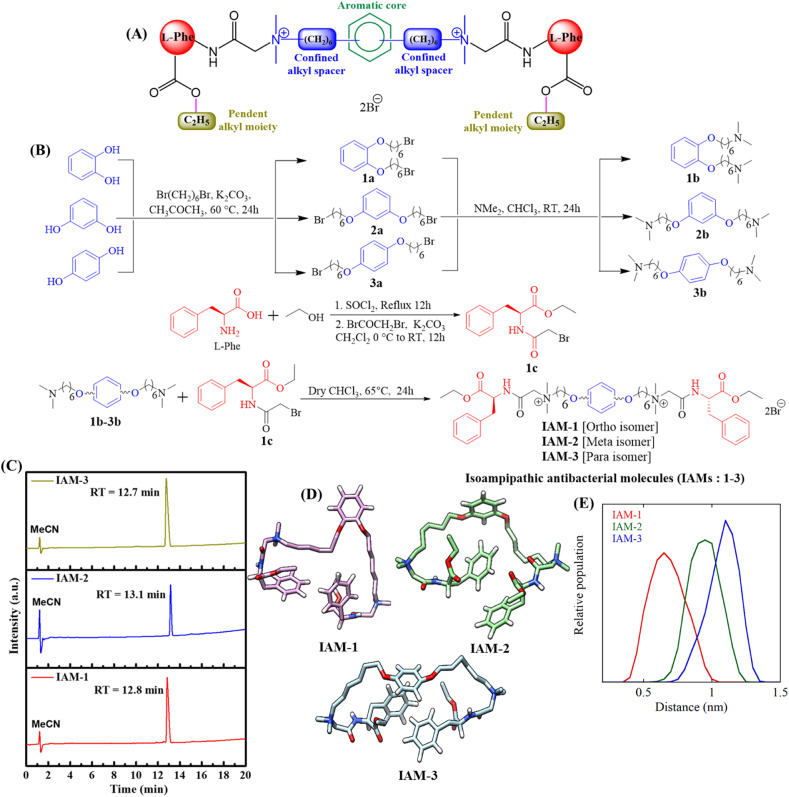
(A) Schematic of molecular design of IAMs; (B) synthesis of isoamphipathic antibacterial molecules (IAMs: 1–3); (C) amphiphilicity of IAMs: 1–3 through HPLC retention time measurement; (D) representative structures obtained from the simulations of the three compounds in aqueous solution (pink, IAM-1, green, IAM-2, and light blue, IAM-3) and (E) distance between the centres of mass of the two C6 alkyl chains, obtained from the simulation.

Towards this goal, herein, we report new isoamphipathic antibacterial molecules (IAMs: 1–3), based on our previous design.^26^ This class of molecules (IAMs) consists of phenylalanine residues, two cationic charges, confined alkyl spacers (in between two positively charged centres), non-peptide amide linkages, and pendant ester functionalities (Fig. 1A). An additional aromatic diol (*ortho*: catechol, *meta*: resorcinol, and *para*: hydroquinone) was incorporated in the confined alkyl spacer region to introduce positional isomerism in the molecular architecture. This resulted in three positional isomers IAM-1 (*ortho*), IAM-2 (*meta*), and IAM-3 (*para*). Herein, pendant ester functionalities were fixed to an ethyl moiety, since they did not exhibit much detrimental effect on mammalian cells, in our previous design of the ASAM series.^26^ In this article, first, molecular dynamics simulations with IAMs: 1–3 were performed to understand the conformation of the isomers in aqueous solution. The antibacterial and hemolytic activities of IAMs 1–3 were evaluated against various bacterial strains and human erythrocytes (hRBCs) showing the importance of positional isomerism in controlling selective interactions with the bacterial envelope over the mammalian membrane. To strengthen the influence of positional isomerism on IAM activity/selectivity, co-culture studies where bacteria and mammalian cells (hRBC RAW cells) were simultaneously present, were conducted. Furthermore, biophysical studies were performed with IAMs 1–3 in model bacterial and mammalian membranes to monitor the change in membrane dynamics/fluidity and the extent of membrane leakage with respect to positional isomerism. Based on the overall biological and biophysical studies, *ortho*-isomer IAM-1 was selected as the lead molecule. Furthermore, bactericidal kinetics, anti-stationary and anti-persister activity, as well as anti-biofilm efficacy of IAM-1, were investigated in detail. In addition, the membrane-active mechanism of bacterial killing was studied with IAM-1, through fluorescence spectroscopy and microscopy studies. Furthermore, the interaction of IAM-1 with model bacterial membranes was studied through molecular dynamics simulations using the minimum bias approach.

## Results and discussion

### Design and synthesis

Our designed molecules are small molecular mimetics of antimicrobial peptides (AMPs), a part of the human innate immune system.^28,29^ In the molecular structure, the central aromatic core, confined alkyl spacers, pendent ester chains, and amino acid side chains contribute as hydrophobic domains. The quaternary amine charges and ether, ester, and non-peptidic amide linkages contribute as hydrophilic counterparts. The core aromatic moiety introduced positional isomerism in the molecular design (Fig. 1A). Herein, isoamphipathic antibacterial molecules (IAMs 1–3) were synthesized by following four simple synthetic steps, as outlined in Fig. 1B. First, aromatic diols (catechol, resorcinol, and hydroquinone) were individually reacted with dibromohexane under mild basic conditions to obtain dibromohexyloxy benzenes (1a, 2a, and 3a). Furthermore, dimethyl amine was reacted with dibromohexyloxy benzene intermediates at room temperature, to produce *N*,*N*,*N*′,*N*′-tetramethyl diaminohexyloxy benzenes (1b, 2b, and 3b). On the other side, the free acid group of l-phenylalanine was esterified under refluxing conditions using ethanol. The crude products originating from the esterification reaction were further reacted with bromoacetyl bromide under basic conditions to obtain the activated ethyl ester bromide derivatives of phenylalanine (1c). To this end, different *N*,*N*,*N*′,*N*′-tetramethyl diaminohexyloxy benzenes (1b, 2b, and 3b) were quaternized with the activated ethyl ester bromide derivatives of phenylalanine (1c) to synthesize all the final molecules (IAMs 1–3) with quantitative yield. All the intermediates and IAMs were characterized thoroughly through ^1^H-NMR, ^13^C-NMR, and HRMS (ESI, Fig. S1–S9†). Importantly, all these isomers (IAMs: 1–3) showed similar retention times (12.7–13.1 min) in HPLC indicating their isoamphipathic nature (Fig. 1C).

Furthermore, to analyse the conformations of the three IAMs in solution, we performed three independent simulations for each compound in water (four in the case of IAM-1), in the presence of a physiological salt concentration, for a total of almost 12 microseconds. Overall, the simulations revealed that all the isomers were extremely flexible and dynamic. Fig. 1D reports the most representative conformation for each of the three compounds. Herein, the different attachment of the two “arms” to the central aromatic moiety determined a different relative arrangement of the confined hexyl chains. In particular, for the *ortho* analogue (IAM-1) the two hexyl groups were on average closer to each other compared to the other two isomers (Fig. 1E).

### Antibacterial and hemolytic activity

**Fig. 2:**
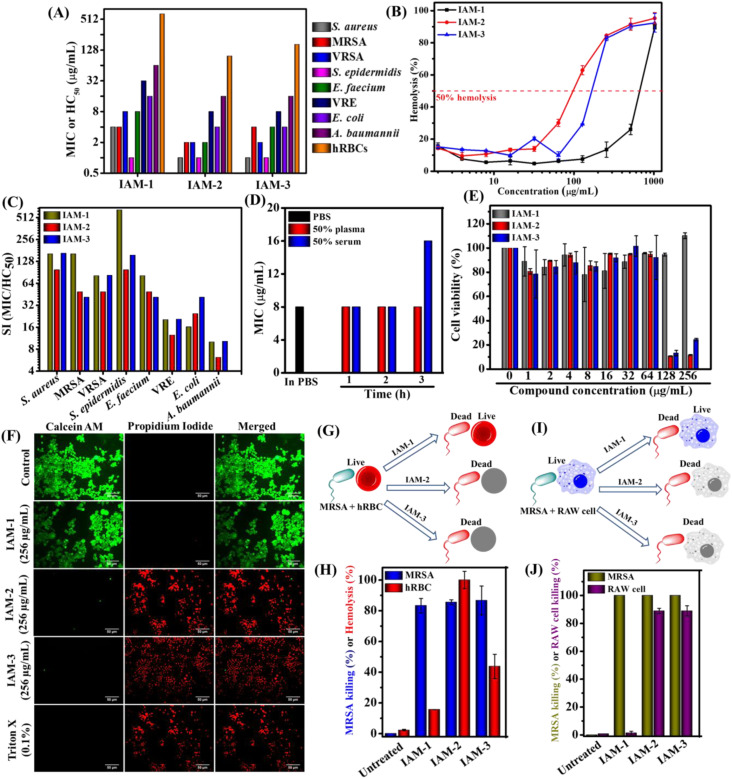
(A) Antibacterial activity (MIC) and toxicity (HC_50_) of IAMs: 1–3. The experiment was performed twice in triplicate; (B) hemolysis percentage upon IAMs: 1–3 exposure at variable concentrations. The experiment was performed twice in triplicate; (C) selectivity index of IAMs: 1–3. The SI values were calculated based on the average values of MIC and HC_50_; (D) antibacterial activity of IAM-1 against MRSA upon human blood plasma and serum pre-incubation at different time intervals (1 h, 2 h and 3 h). The experiment was conducted twice in triplicate; (E) RAW cell viability upon IAMs: 1–3 treatment; (F) fluorescence microscopy of RAW cells upon IAMs: 1–3 treatment at 256 μg mL^−1^ (257.8 μM); (G) schematic of a MRSA and hRBC co-culture study; (H) co-culture of MRSA and hRBC in the presence and absence of IAM-1, IAM-2 and IAM-3 at 256 μg mL^−1^ (257.8 μM); (I) schematic of a MRSA and RAW cell co-culture study and (J) co-culture of MRSA and RAW cells in the presence and absence of IAM-1, IAM-2 and IAM-3 at 256 μg mL^−1^ (257.8 μM). # MRSA: methylene resistant *S. aureus*, VRSA: vancomycin resistant *S. aureus* and VRE: vancomycin resistant *E. faecium*.

The antibacterial activity and hemolytic activity of all the IAMs 1–3 were evaluated against various Gram-positive and Gram-negative bacteria including multi-drug resistant clinical isolates and human red blood cells (hRBCs) respectively through the broth dilution method (Fig. 2A and ESI Table 1†). The antibacterial activity was expressed in terms of minimum inhibitory concentration (MIC) (concentration of the test compound required for complete inhibition of bacterial growth). Hemolytic activity was presented as HC_50_ (concentration of the test compound accountable for 50% lysis of hRBCs). The isomers displayed moderate to good antibacterial activity [*ortho* isomer: IAM-1; MIC = 1–64 μg mL^−1^ (1–64.4 μM), *meta* isomer: IAM-2; MIC = 1–16 μg mL^−1^ (1–16.1 μM) and *para* isomer: IAM-3; MIC = 1–16 μg mL^−1^ (1–16.1 μM)]. Upon testing against Gram-positive bacteria (*S. epidermidis*, *S. aureus*, MRSA, VRSA, and *E. faecium*), all three positional isomers, IAMs 1–3 completely inhibited bacterial growth at almost similar concentrations with the MIC ranging from 1–8 μg mL^−1^ or μM. Importantly, all these isomers were active against Gram-negative bacterial species. In the case of *E. coli*, the compounds exhibited MIC values in the range of 4–16 μg mL^−1^ (4–16.1 μM). Although these isomeric molecules (IAMs 1–3) did not display a drastic difference in antibacterial activity, they showed a remarkable differentiation with respect to the hemolytic activity. The *ortho* isomer, IAM-1 exhibited a much higher HC_50_ value of 650 μg mL^−1^ (654.6 μM) compared to *meta*-isomer, IAM-2 [HC_50_ = 98 μg mL^−1^ (98.7 μM)], and *para*-isomer, IAM-3 [HC_50_ = 160 μg mL^−1^ (161.1 μM)] (Fig. 2A and ESI Table 1†). At a concentration of 256 μg mL^−1^ (257.8 μM) (*i.e.* much higher than the MIC values), the extent of hRBC lysis for IAM-1 was only 13% whereas for IAM-2 and IAM-3 it was >80% (Fig. 2B). This suggested that the toxicity profile rather than the antibacterial activity of phenylalanine and hexyl bearing compounds, IAMs 1–3 was strongly influenced by positional isomerism. Furthermore, to understand the impact of positional isomerism, the selectivity index (HC_50_/MIC) was calculated for IAMs 1–3 against all the tested Gram-positive and Gram-negative bacteria. Fig. 2C reveals that IAM-1 showed 650 fold, 162 fold, and 81 fold selectivity against *S. epidermidis*, MRSA and *E. faecium* respectively over human erythrocytes. In contrast, 98–160 fold, 40–49 fold, and 41–49 fold selectivity against *S. epidermidis*, MRSA, and *E. faecium* respectively were displayed by the other two isomers, IAM-2 and IAM-3 over human erythrocytes. In the cases of *S. aureus*, VRSA, *E. coli,* and *A. baumannii*, the *ortho* (IAM-1) and *para* (IAM-2) isomers showed similar selectivity while *meta*- (IAM-3) isomer's selectivity was quite low. Altogether, all three isomers showed a good selectivity index. More interestingly, the *ortho* isomer, IAM-1 showed superior selectivity towards bacteria over mammalian cells in comparison to the *meta* and *para* isomers. Based on the overall structure activity relationship (SAR), IAM-1 was considered the optimized molecule for further investigation of its antibacterial efficacy in detail. Most importantly, IAM-1, IAM-2, and IAM-3 were taken forward for the comparative studies on the role of positional isomerism in controlling selective interaction with bacteria over mammalian membranes.

### Plasma and serum stability

Antibacterial compounds immune to instantaneous protease (present in blood plasma and serum) degradation unlike natural antimicrobial peptides (AMPs) have significant clinical relevance. Therefore, we evaluated the antibacterial activity (MIC) of the lead compound, IAM-1 against MRSA upon pre-incubation with 50% human blood plasma and serum at different time intervals (1 h, 2 h, and 3 h) (Fig. 2D). Interestingly, the plasma pre-incubation led to no loss of antibacterial activity of IAM-1. Similarly, no changes in the MIC of IAM-1 were observed upon 1 h and 2 h serum pre-incubations. However, 3 h serum pre-incubation of IAM-1 resulted in a slight increase in its MIC by 2 fold (Fig. 2D). The result altogether demonstrated that this class of compound is relatively stable under complex blood components.

### Cytotoxicity

Furthermore, the toxicity of the three positional isomers (IAMs: 1–3) was evaluated by studying the cell viability against RAW cell lines through Alamar blue assay (Fig. 2E). The *ortho* isomer (IAM-1) showed 100% cell viability at 256 μg mL^−1^ (257.8 μM) after treating RAW cells for 24 h. However, *meta*-(IAM-2) and *para*-(IAM-3) exhibited 8% and 24% cell viability at 256 μg mL^−1^ (257.8 μM).

In addition to this study, fluorescence microscopy of RAW cells was performed upon IAMs 1–3 treatment by simultaneous staining with calcein-AM (stains live cells with green emission) and PI (stains cells with compromised cell membranes with red emission) (Fig. 2F). All the cells were found to be alive in the presence of IAM-1 [256 μg mL^−1^ (257.8 μM)]. Nevertheless, almost no live cells were witnessed upon IAM-2 (*meta*-isomer) and IAM-3 (*para*-isomer) treatment at 256 μg mL^−1^ (257.8 μM). The overall results further ensured the superiority of the *ortho*-isomer over the *meta*- and *para*-isomers in terms of their detrimental effect on mammalian cells.

### Co-culture study

To strengthen the impact of positional isomerism in achieving selective interaction, bacterial (MRSA) and mammalian cells (hRBCs or RAW cells) were treated with IAM 1–3 [256 mg mL^−1^ (257.8 μM)] under co-culture conditions instead of treating them individually^32^ (Fig. 2F–I). A co-culture of MRSA and human erythrocytes (hRBCs) revealed 83% killing of MRSA and 16% lysis of hRBCs by the *ortho*-isomer, IAM-1 (Fig. 2G and H). In contrast, the *meta*-isomer, IAM-2 displayed 85% killing of MRSA and 100% lysis of hRBCs. Similarly, the *para*-isomer (IAM-3) exhibited 86% killing of MRSA and 44% lysis of hRBCs (Fig. 2G and H). On the other hand, 100% and 1.4% killing of MRSA and RAW cells, respectively, were observed upon 3 h of treatment with the *ortho*-isomer, IAM-1 under co-culture conditions (Fig. 2I and J). In contrast, the *meta*- (IAM-2) and *para*-(IAM-3) isomers equally eliminated both MRSA (100% killing) and RAW cells (89% killing). However, the co-culture conditions did not affect the survival of both bacterial and mammalian cells in the absence of compound treatment in the case of untreated controls within the experimental time frame (15 min for hRBCs and 3 h for RAW cells). These results indicated that the *ortho*-isomer selectively killed bacteria over mammalian cells. However, *meta*- and *para*-isomers were equally detrimental to both bacteria and mammalian cells at the tested concentration. Overall, this result demonstrated the impact of positional isomerism in achieving selective antibacterial agents without altering the overall hydrophobicity of the molecular design.

### Membrane leakage

**Fig. 3:**
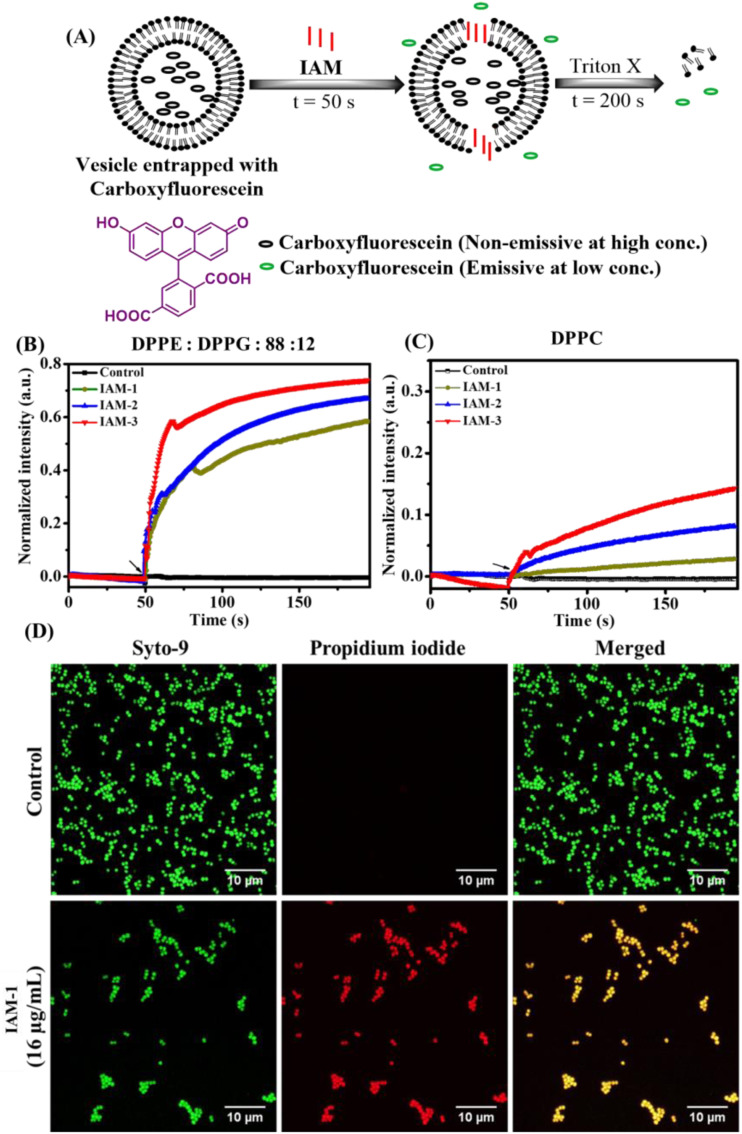
(A) Schematic of a membrane leakage study using the carboxyfluorescein dye; (B) bacterial model membrane (DPPE : DPPG: 88 : 12) leakage upon treatment with IAMs: 1–3 at 160 μg mL^−1^ (161.1 μM); (C) mammalian model membrane (DPPC) leakage upon treatment with IAMs: 1–3 at 200 μg mL^−1^ (201.4 μM). The black arrows indicated the time of compound addition to the vesicles and (D) investigation on the antibacterial mechanism of action with the lead compound, IAM-1: through fluorescence microscopy of MRSA upon IAM-1 treatment for 6 h.

The impact of positional isomerism on membrane perturbation was also studied by monitoring the leakage of carboxyfluorescein dye encapsulated within the liposomes made of DPPG : DPPE (88 : 12) and DPPC lipids mimicking bacterial and human erythrocyte model membranes, respectively (Fig. 3A–E). Specifically, carboxyfluorescein does not show significant emission when it is trapped within liposomes/vesicles, due to self-quenching effects at concentrations higher than 30 mM.^32^ On the other hand, this particular dye displays a strong emission under diluted conditions (3 mM) when it releases from the vesicles upon interacting with an external membrane perturbing agent (Fig. 3A). Herein, dye-trapped vesicles were treated with IAMs 1–3 [160 μg mL^−1^ (161.1 μM)] (*ortho*-, *meta*- and *para*-isomers) at 50 s and the change in emission intensity maxima of carboxyfluorescein was monitored for 200 s (Fig. 3A). In the case of the bacterial model membrane, fluorescence intensity was enhanced upon treatment with all the positional isomers (IAMs 1–3) indicating membrane leakage or membrane perturbation (Fig. 3B). More importantly, almost no enhancement in fluorescence intensity was observed upon the treatment of the mammalian model membrane with the *ortho*-isomer, IAM-1, indicating no significant membrane leakage (Fig. 3B).

In contrast, *meta*-(IAM-2) and *para*-(IAM-2) isomers displayed substantial enhancement in fluorescence intensity upon interacting with the mammalian model membrane (Fig. 3C). This suggested that *meta*- and *para*-isomers had a profound effect on the mammalian membrane along with the bacterial membrane. Furthermore, the rate constant of dye leakage was evaluated by fitting the data points of the emission spectrum [between compound addition (at 50 s) and triton-X addition (at 200 s)] using an exponential equation (Fig. S10†). Interestingly, the rate constant (*k*) of dye leakage was ∼10 times higher in the case of the bacterial model membrane (*k* = 1.9 × 10^−2^ s^−1^) compared to the mammalian model membrane (*k* = 3 × 10^−3^ s^−1^) in the case of the *ortho*-isomer (IAM-1). However, the rate constant was of a similar magnitude for both bacterial and mammalian model membrane upon treatment with *meta*-[IAM-2; *k* (bacterial membrane): 1.9 × 10^−2^ s^−1^ and *k* (mammalian membrane): 1 × 10^−2^ s^−1^] and *para*-[IAM-3; *k*(bacterial membrane): 3 × 10^−2^ s^−1^ and *k* (mammalian membrane): 0.8 × 10^−2^ s^−1^] isomers. The results altogether established that the *ortho*-isomer was superior at the tested concentration in comparison to the *meta*- and *para*-isomers in achieving selective interaction with bacterial membranes over mammalian membranes.

Furthermore, to study the association of IAM-1 with the mammalian and bacterial model phospholipid membranes composed of POPC and cholesterol (1 : 1) or POPE/POPG (7 : 3) respectively, the compound's absorbance and emission properties were exploited. The normalized absorbance and emission spectra of IAM-1 in an aqueous solution are dominated by the dialkoxybenzene moiety, with only minor contributions by the phenylalanine residues (Fig. S11A†).^33^ Herein, a fixed IAM concentration (10 μM) was titrated with increasing concentrations of vesicles. Membrane association caused an increase in fluorescence intensity, from which we could determine the fraction of membrane-bound compounds (Fig. S11B and S11C†). The water-membrane partition curves clearly show a much stronger affinity of IAM-1 for liposomes mimicking the composition of bacterial membranes compared to the mammalian membrane. The anionic charge of POPG lipids definitely plays a role in the observed selectivity, but the negative intrinsic curvature of POPE and the effects of cholesterol on lipid packing likely contribute too.^34^

Furthermore, the influence of positional isomerism was established also by studying the change in membrane fluidity/dynamics upon compound treatment by encapsulating laurdan dye (6-dodecanoyl-2-dimethylaminonaphthalene) inside bacterial and mammalian model membranes (ESI Figure S12†).^30,31^

### Membrane active mechanisms

**Fig. 4:**
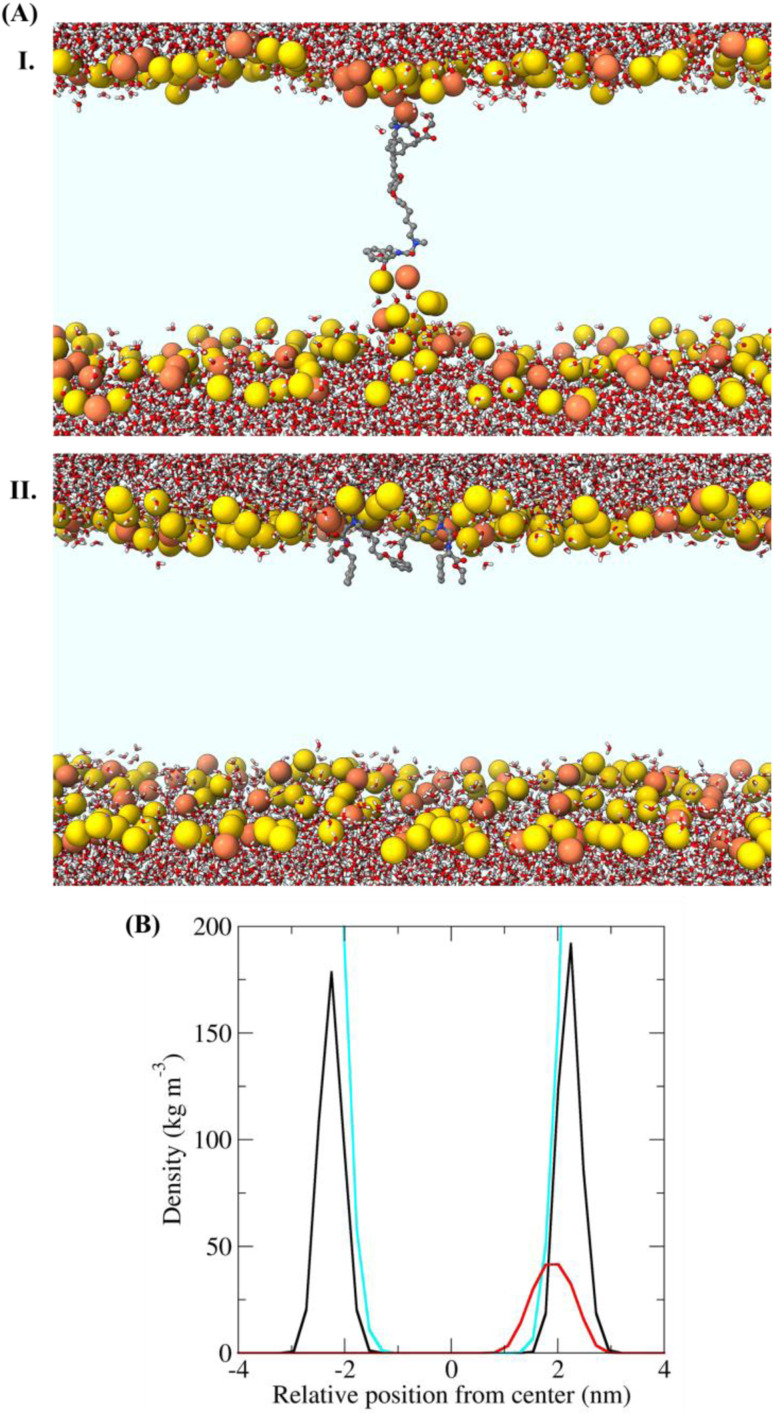
(A) Structures of the bacterial membrane in the three simulations in which a defect free bilayer was formed. Water molecules and IAM-1 are shown in stick representation and coloured according to atom type (C = grey, O = red, N = blue, and H = white). The phosphorus atoms of the lipids are shown as yellow or orange spheres (for POPE and POPG, respectively). (I) Transmembrane orientation and (II) superficial orientation of IAM-1 in the lipid bilayer. The other parts of the lipid molecules are not shown, for the sake of clarity, so that the light blue area corresponds to the hydrophobic core of the bilayer. (C) Density of selected groups in the equilibrated trajectory (last 20 ns, after the annealing process), showing the different depth of insertion of the analogue. The colour code used in the structural figures is the same as that in Fig. 4B and water is not shown, for the sake of clarity. In the bottom panel the density is shown in black for P atoms, in cyan for water and in red for IAM-1 respectively.

The biophysical investigation on membrane fluidity and membrane leakage as described previously indicated the membrane active nature of isoamphipathic antibacterial molecules. However, the detailed membrane activity of the lead *ortho*-isomer (IAM-1) was further investigated by performing membrane depolarization and outer membrane permeabilization against MRSA and *E. coli* respectively (Fig. S13A and S13B†). The membrane active nature was further supported by studying fluorescence microscopic analysis of bacteria upon IAM-1 treatment (Fig. 3D). Finally, the interaction of the compound with bacterial membranes was investigated through a theoretical study using the minimum bias approach (Fig. 4A and B).

### Membrane depolarization

Membrane-potential sensitive dye 3,3′-dipropylthiadicarbocyanine iodide [DiSC3 (5)] was selected for this study. Under normal potential across the membrane, DiSC3 (5) dye distributes inside and outside the bacterial envelope. Thereby, the fluorescence intensity of the dye decreases, due to its self-quenching inside the bacterial cells. Treatment with compounds which affect the normal membrane potential results in the release of the dye in the external media, leading to an increase in fluorescence intensity.^27^ Due to the addition of various concentrations of IAM-1 [16 μg mL^−1^ (16.1 μM) and 32 μg mL^−1^ (32.2 μM)] an increment in fluorescence intensity was observed for both *S. aureus* and *E. coli* (Fig. S13A†). The extent of fluorescence enhancement followed a concentration dependence (greater effect at higher concentrations of the compound). This observation clearly indicated the perturbation of normal membrane potential by this class of molecules.

### Outer membrane (OM) permeabilization

Since the outer membrane (OM) of Gram-negative bacteria serves as a diffusion barrier for multiple antibiotics, the OM permeabilization of IAM-1 was investigated against *E. coli* using hydrophobic dye *N*-phenyl naphthylamine (NPN). This particular dye emits upon binding to the hydrophobic region of the bacterial outer membrane when an external membrane active agent perturbs it.^27^ Herein, NPN fluorescence intensity was observed to be enhanced upon treatment with IAM-1 at different concentrations [32 μg mL^−1^ (32.2 μM) and 64 μg mL^−1^ (64.4 μM)] (Fig. S13B†). The extent of fluorescence enhancement followed a concentration dependence (greater effect at higher concentrations of the compound). These results confirmed the outer membrane perturbation ability of the compound in a dose-dependent mode.

### Fluorescence microscopy

The membrane active nature of IAM-1 was further confirmed by performing fluorescence microscopy of MRSA by concomitant staining with Syto-9 and PI (Fig. 3D). In the case of the untreated control, all the MRSA cells were alive since they were solely stained with green emissive Syto-9 dye. Importantly, IAM-1 [16 μg mL^−1^ (16.1 μM)] treated MRSA cells were equally stained with both green emissive Syto-9 and red emissive PI and the merged image showed a co-localization of both dyes. This result indicated that the bacterial cell membranes were compromised upon compound treatment and re-established the membrane active nature of IAM-1.

### Molecular dynamics simulations

To further support the membrane active nature, we performed simulations of the lead compound, IAM-1 in the presence of POPE/POPG lipids, using the so-called “minimum-bias” approach, where simulations start from a random mixture of bioactive molecules, lipids, and water. With this approach, the bilayer self-assembles, and the system is initially very fluid. This method can individuate the positions and orientations of the molecule that correspond to free energy minima.^35^ Since our goal was to determine the position, orientation, and conformation of the IAM in the membrane-bound state, we simulated conditions under which pore formation was not expected (IAM-1/lipid = 1/128). Three independent simulations were performed for IAM-1. A defect-free bilayer formed during the simulation time only in two cases. Representative structures at the ends of these trajectories are reported in Fig. 4A. IAM-1 populated both a superficial and a transmembrane orientation. IAM-1 inserted below the head groups when surface bound. It adopted an M-shaped conformation, with the phenyl and alkoxybenzene groups pointing towards the hydrophobic core of the bilayer and the cationic amino groups interacting with the phosphates of the lipids (Fig. 4B). The structure of the IAM-1 inserted in the transmembrane orientation shows that this molecule cannot reach easily from one side to the other of the membrane. In the final part of the trajectory, the distance between the two N atoms of the charged amino groups was on average 1.8 nm, *i.e.*, less than the normal thickness of the bilayer (approximately 4 nm). As a consequence, a local thinning of the membrane was observed.

Overall, these data suggest that the IAM molecules might act similarly to antimicrobial peptides, by binding to the membrane surface, below the head groups, and thus inducing stress in the surface tension of the outer lipid leaflet.^34,35^ This stress, in turn, can lead to IAM insertion in a transmembrane orientation and/or to membrane leakage or membrane perturbation.

### Time-kill kinetics

**Fig. 5:**
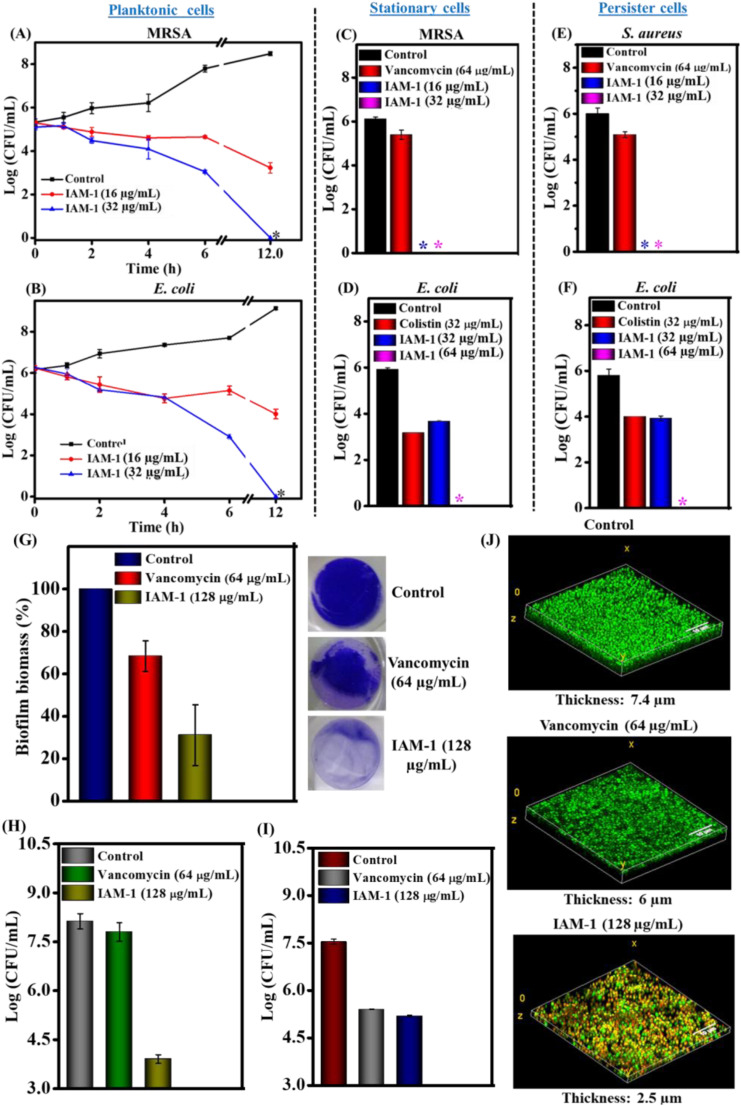
Bactericidal kinetics of IAM-1 against planktonic (A) MRSA ATCC 33591 and (B) *E. coli* MTCC 443; anti-stationary phase activity of IAM-1 (upon 6 h treatment) against stationary cells of (C) MRSA ATCC 33591 and (D) *E. coli* MTCC 443; anti-persister phase activity of IAM-1 against persister cells of (E) *S. aureus* MTCC 737 and (F) *E. coli* MTCC 443. The asterisk (*) corresponds to < 50 CFU. MRSA biofilm disruption upon IAM-1 treatment: (G) crystal violet staining of MRSA ATCC 33591 biofilm; (H) biofilm-embedded MRSA ATCC 33591 cell viability; (I) biofilm-dispersed MRSA cell viability and (J) fluorescence microscopy of MRSA ATCC 33591 biofilm disruption. The live/dead ratio; control: 96% : 4%, vancomycin [64 μg mL^−1^ (44.2 μM)]: 80% : 20% and IAM-1 [128 μg mL^−1^ (128.9 μM)]: 15% : 75%. The live/dead ratio was calculated using image J.

To understand the antibacterial action window, bactericidal kinetics studies of the lead compound, IAM-1 were performed against planktonic MRSA and *E. coli* (Fig. 5A and B). IAM-1 at 16 μg mL^−1^ (16.1 μM) reduced 2 log of MRSA and *E. coli* respectively at 12 h. Importantly, at a higher concentration of 32 μg mL^−1^ (32.2 μM) both MRSA and *E. coli* were completely (5–6 log reduction) eliminated by IAM-1 at 12 h. These observations suggested that the lead *ortho*-isomer displayed relatively delayed bactericidal kinetics in a dosage dependent manner.

### Activity against metabolically inactive bacteria

Parallel with antimicrobial resistance, the predominance of metabolically inactive bacterial sub-population in chronic infections is another major challenge to global public health.^36,37^ Stationary and persister phase bacterial species belong to these metabolically inactive dormant sub-classes. Most of the conventional antibiotic therapeutics are ineffective against dormant bacteria since they almost shut down most of their biological processes (*e.g.* cell division, protein synthesis, DNA replication, *etc.*), a specific target of the antibiotics. Hence, the anti-stationary and anti-persister activity of the lead *ortho*-isomer (IAM-1) was investigated as depicted in Fig. 5C–F. For instance, IAM-1 at both 16 μg mL^−1^ (16.1 μM) and 32 μg mL^−1^ (32.2 μM) completely (6log reduction) eradicated stationary cells of MRSA (Fig. 5C). Similarly, at a lower concentration of 32 μg mL^−1^ (32.2 μM), IAM-1 showed 2.2 log reduction of stationary cells of *E. coli.* Noticeably, complete elimination of *E.coli* stationary cells was observed at a twofold higher concentration of 64 μg mL^−1^ (64.4 μM) (Fig. 5D). Alongside, IAM-1 killed the persister cells of *S. aureus* completely (6 log reduction) at both 16 μg mL^−1^ (16.1 μM) and 32 μg mL^−1^ (32.2 μM) (Fig. 5E). Likewise, the compound displayed 2 log reduction of *E. coli* persister cells at 32 μg mL^−1^ (32.2 μM). Moreover, the compound was efficacious in the complete elimination of *E. coli* persister cells at 64.4 μM (Fig. 5F). However, the widely prescribed antibiotic for Gram-positive infection, vancomycin [64 μg mL^−1^ (64.4 μM)] displayed negligible reduction (0.5–1 log reduction) of stationary and persister cells of MRSA and *S. aureus*. On the other hand, colistin, a widely used antibiotic for Gram-negative infection, [32 μg mL^−1^ (27.7 μM)] displayed 2 log and 3 log reduction of stationary and persister cells of *E. coli* respectively. Taken together, these data demonstrated that the lead isoamphipathic antibacterial molecule (IAM-1) was competent enough to combat dormant bacteria, one of the critical factors behind chronic and recurrent infections. Remarkably, this class of antibacterial molecules is way better than conventional antibiotics at tackling dormant bacterial sub-populations.

### Anti-biofilm efficacy

An escalation tendency of biofilm formation by multidrug-resistant bacteria is one of the emerging problems within the hurdles of antimicrobial resistance.^5,6,38,39^ Almost 80% of the infections are in conjunction with biofilm formation.

The abundance of thick extracellular polymeric matrices accompanied by dormant bacterial sub-populations in the biofilm renders conventional antibiotics ineffective. Hence, antimicrobials with potent anti-biofilm efficacy are in high demand. Towards this aim, the biofilm disruption efficacy of IAM-1 was investigated through crystal violet (CV) staining and bacterial cell viability counting (Fig. 5G and H). IAM-1 displayed ∼70% reduction in the MRSA biofilm biomass at 128 μg mL^−1^ (128.9 μM) (32 × MIC) in CV staining (Fig. 5G). Positively charged IAM-1 interacts with various negatively charged components of extracellular polymeric matrices of the biofilm and hydrophobicity of the compound aids in biofilm disruption. However, 30% reduction in biofilm biomass was observed upon vancomycin treatment at its 64 times MIC [64 μg mL^−1^ (64.4 μM)]. Additionally, bacterial cell viability assay was performed to understand whether the biofilm-embedded MRSA was killed by the compound along with biofilm disruption. IAM-1 at 128 μg mL^−1^ (128.9 μM) killed 3.5 log of biofilm-trapped MRSA whereas vancomycin at 64 μg mL^−1^ (44.2 μM) was completely ineffective in eliminating MRSA in the biofilm (Fig. 5H). Furthermore, the efficacy of the compound against biofilm-dispersed bacterial cells was investigated. IAM-1 (128.9 μM) displayed 2.3 log reduction (99.5%) of bacterial burden originating from biofilm dispersion (Fig. 5I). In addition, biofilm disruption was visualized through fluorescence microscopy by dual staining with Syto-9 (stains both live and dead cells with green emission) and PI (stains membrane compromised bacterial cells with red emission). Treatment with IAM-1 at 128 μg mL^−1^ (128.9 μM) drastically reduced the thickness of the MRSA biofilm to 2.5 μm while the untreated biofilm displayed a thickness of 7.4 μm (Fig. 5J). Along with the thickness reduction, the compound was competent to kill biofilm-embedded MRSA ensured by the presence of a large fraction of MRSA (75% dead) with yellow staining (due to colocalization of green and red colours). In contrast, upon vancomycin [64 μg mL^−1^ (44.2 μM) treatment, the biofilm showed green emission (80% live MRSA) with a thickness of 6 μm. In this context, we hypothesize that probably the interaction between positively charged IAM-1 and various negatively charged components of extracellular polymeric matrices of the biofilm is responsible for biofilm disruption. Additionally, efficacy against dormant bacterial sub-population can be held accountable for the killing of biofilm-embedded MRSA by IAM-1. These overall results suggested that the lead *ortho*-isomer, IAM-1 was a promising candidate with a potent biofilm disruption efficacy.

### Dermal toxicity

**Fig. 6:**
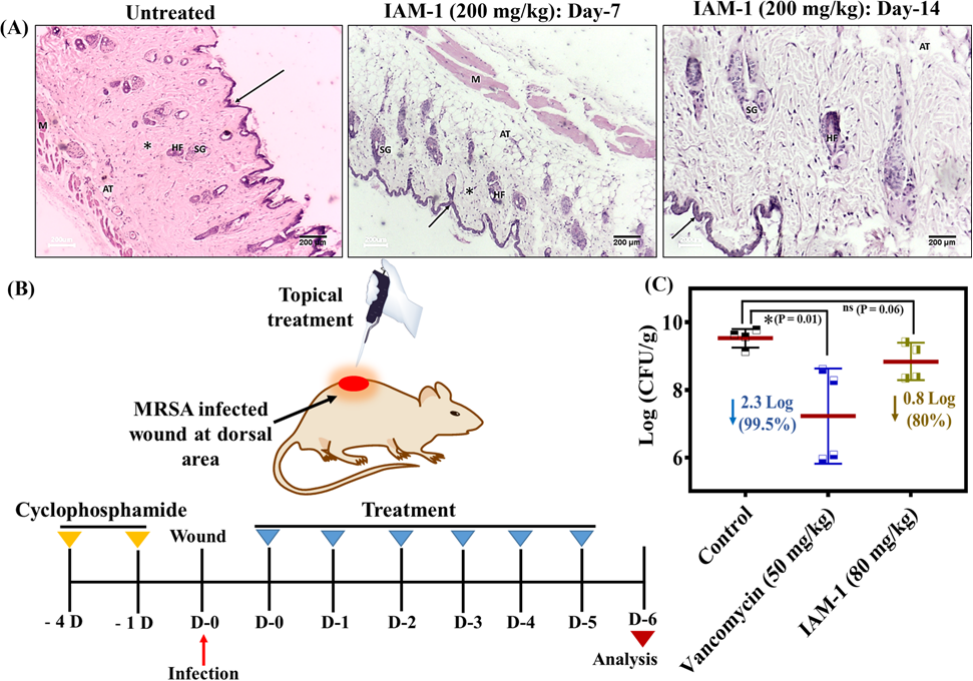
(A) Histopathology (H and E staining) of untreated and IAM-1 (200 mg kg^−1^) treated mouse dermal tissue; wound infection with MRSA, (B) experimental plan and (C) experimental outcome. Values are presented as mean ± standard deviation for a group of 4 mice. Statistical analysis was conducted using GraphPad Prism 7 through an unpaired student-*t* test and *P* < 0.05 was considered statistically significant.

The therapeutic application of the lead compound requires toxicological evaluation in animal models as a part of pre-clinical studies. Therefore, the dermal toxicity of IAM-1 was evaluated upon its topical application at 200 mg kg^−1^ to the dermal layers in the dorsal area of the mice. The effect of the compound on the mice's skin surface was monitored visually for 14 days. We did not observe any significant side effects (such as convulsions, tremors, salivation, irritation, *etc.*) of the compound on the mice. Furthermore, the histopathological changes were investigated through hematoxylin and eosin staining of both saline treated and compound treated dermal tissues (Fig. 6A). The saline treated dermis showed the normal appearance of an epidermis layer lined with stratified squamous epithelial cells and covered by keratin layers (arrow). The dermis contained the sweat and sebaceous glands (SGs), hair follicles (HFs), adipose tissue, and muscle layer (M). The dermis also showed the presence of a spindle-shaped fibrous connective layer with an oval-shaped nucleus (asterisk). The compound treatment also led to a similar observation on day 7 and day 14 (Fig. 6A). These results suggested the non-toxic nature of the compound in the animal model and revealed its suitability for *in vivo* topical application.

### *In vivo* antibacterial activity

Furthermore, the antibacterial efficacy of IAM-1 was evaluated in a murine model of dermal wound infection against MRSA. A wound was created using a scalpel in the dorsal area of neutropenic mice after fur removal and the wound was infected with 10^7^ CFU mL^−1^ inoculation of MRSA. Four hours post-infection, the wound was treated topically with 80 mg kg^−1^ (below the tolerable dosage of dermal administration) IAM-1, and 50 mg kg^−1^ vancomycin treatment was also initiated as a positive control. The same dosages of treatments (OD: once a day) were continued with vancomycin and compound for 5 consecutive days (Fig. 6B). Furthermore, bacterial cell viability assay within the infected dermal tissues was conducted on the 6th day. The results demonstrated a 2.3 log reduction (99.5%) of MRSA burden by vancomycin (Fig. 6C). Similarly, IAM-1 displayed an 80% reduction in MRSA burden compared to the untreated control (Fig. 6C).

## Conclusion

A new class of amino acid based isoamphipathic antibacterial molecules has been developed and they showed appreciable antibacterial activity against various Gram-positive and Gram-negative bacteria including multi-drug resistant clinical isolates. More importantly, apart from hydrophobicity modulation in controlling antibacterial activity and toxicity, positional isomerism has been recognized as an influential factor in the design. Co-culture studies, investigation on membrane dynamics through molecular dynamics (MD) simulations and membrane leakage and membrane fluidity studies established the superiority of the *ortho*-isomer (IAM-1) in achieving selective interaction with bacterial membranes over mammalian membranes in comparison to *meta*-(IAM-2) and *para*-(IAM-3) isomers owing to its local membrane thinning effect. MD simulation, biophysical and spectroscopic studies also revealed that this class of compound primarily targets bacterial membranes through different modes of action such as transmembrane orientation, membrane leakages or perturbation *etc.* Moreover, the potency to combat metabolically inactive bacterial sub-population and bacterial biofilms made the *ortho*-isomer a promising candidate to encounter chronic infections. The lead isomer displayed moderate *in vivo* antibacterial activity in a murine model of wound infection against MRSA with no notable *in vivo* dermal toxicity. Altogether, the current article explored a new guiding parameter for achieving a selective antibacterial agent.

## Ethical statement

The antibacterial studies including hemolytic activity were conducted as per the approval of the Institutional Bio-safety Committee of the Jawaharlal Nehru Centre for Advanced Scientific Research (JNC/IBSC/2020/JH–12). The *in vivo* animal studies were executed according to the protocols sanctioned by the Institutional Animal Ethics Committee (IAEC) of the Jawaharlal Nehru Centre for Advanced Scientific Research (JNCASR) (201/Go/ReBi/S/2000/CPCSEA).

## Data availability

The synthetic protocols for IAMs, molecular characterization, experimental protocols for biological, biophysical, and animal studies, and theoretical studies can be found in the ESI.†

## Author contributions

The work and the experiments were designed by S. B. and J. H. The theoretical studies were designed, performed, and analyzed by A. G., P. C., G. B., and L. S. Cell toxicity assay was performed by B. B. All other studies were conducted by S. B., S. M., L. J, and D. B. All the data were analyzed by S. B., S. M., and J. H. The membrane leakage results were analyzed by D. B., S. B. and J. H. The water partition to membrane study was conducted and analyzed by C. T. and L. S. The manuscript was written by S. B., J. H., and L. S. All the authors approved the final version of the manuscript.

## Conflicts of interest

The authors declare no conflicts of interest.

## Supplementary Material

SC-014-D2SC06065E-s001
