# Supplementary material for: Isoamphipathic antibacterial molecules regulating activity and toxicity through positional isomerism
Source: Chem Sci. 2023 Apr 6;14(18):4845–56. doi: 10.1039/d2sc06065e (PMC10171078; doi:10.1039/d2sc06065e)
Supplement: SC-014-D2SC06065E-s001 [file SC-014-D2SC06065E-s001.pdf]

Electronic Supplementary Material (ESI) for Chemical Science.
This journal is © The Royal Society of Chemistry 2023

Supporting Information

Isoamphipathic Antibacterial Molecule Regulating Activity and Toxicity through Positional Isomerism

Swagatam Barman,[a] Sudip Mukherjee,[a] Logia Jolly,[a] Cassandra Troiano,[b] Alessandro Grottesi,[c] Debajyoti Basak,[a] Paolo Calligari,[b] Brinta Bhattacharjee,[a] Gianfranco Bocchinfuso,[b] Lorenzo Stella,[b] and Jayanta Haldar*[a,d]

[a] Antibacterial Research Laboratory, New Chemistry Unit, Jawaharlal Nehru Centre for Advanced Scientific Research (JNCASR), Jakkur, Bangalore, India-560064. E-mail: jayanta@jncasr.ac.in.

[b] Department of Chemical Science and Technologies, University of Rome Tor Vergata, via della Ricerca Scientifica, 1; 00133, Rome, Italy.

[C]Cineca, Via dei Tizii, 6; 00185, Rome, Italy

[d] School of Advanced Materials, Jawaharlal Nehru Centre for Advanced Scientific Research (JNCASR), Jakkur, Bangalore, India-560064

Table of Contents:

Page S2-S14: Synthetic protocol and experimental protocols

Page S14-S23: Supplementary figures

Experimental Section

Materials and instrumentations. Dichloromethane, ethanol, thionyl chloride was obtained from Spectrochem (India) and all the solvents were of reagent grade and dried before use according to the requirement. Bromoacetyl bromide, 1,4-dibromohexane, 1,6-dibromooctane, 1,10-dibromodecane, catechol, hydroquinone, resorcinol, *N*-phenyl naphthylamine, propidium iodide, 3, 3′-dipropylthiadicarbocyanine iodide [$DiSC_3$ (5)], DPPE, DPPG, DPPC, 5(6)-carboxyfluorescein, laurdan dye were procured from Sigma-Aldrich. L-Phenylalanine was obtained from Spectrochem (India). Analytical thin layer chromatography (TLC) was performed on silica gel 60 F254 percolated E. Merck TLC plates and visualized by using iodine or ninhydrin. Cholesterol, POPC, POPG and POPE were purchased from Avanti Polar Lipids (Birmingham, AL, USA) with a purity >99%. Bruker AMX-400 spectrometer was used to record nuclear magnetic resonance (NMR) spectra in deuterated solvents. 6538-UHD Accurate Mass Q-TOF LC-MS instrument was used to record mass spectra. Tecan Infinite M200 PRO Microplate Reader was used for optical density (O.D.) measurement. UV-visible experiments were performed on a Jasco V-770 spectrophotometer. Fluorescence measurements for the biophysical investigations were carried out using Jasco FP-8500 spectrofluorimeter. Bacterial strains, *S. aureus* MTCC737, *E. coli* MTCC443, *A. baumannii* MTCC1425 were procured from MTCC (Chandigarh, India). VRE 909 and VRSA 4 were obtained from Anthem Bioscience (Bangalore, India). MRSA ATCC33591, *E. faecium* ATCC19634, and *K. pneumoniae* ATCC700603 were purchased from ATCC (Rockville, MD, USA). *S. aureus*, *E. coli*, MRSA, *K. pneumoniae* and *A. baumannii* were grown in Mueller Hinton Broth (MHB-HIMEDIA-M391). Brain heart infusion broth (BHI) was to inoculate *E. faecium* and VER. MacConkey Agar and Nutrient agar were used as a solid media for Gram-negative and gram-positive bacteria. 96 well plates, 6 well plates and transparent black 96 well plates were obtained from Vasa Scientific (Bangalore, India).

Synthesis and characterization

Synthetic protocol of dibromohexyloxy benzene (1a, 2a and 3a). Aromatic diol (catechol / resorcinol / hydroquinone) (2.5 g, 22.7 mmol), *N*, *N′*-hexyl dibromide (10.5 mL, 68 mmol) and potassium carbonate (14.2 g, 102.7 mmol) were taken together in a RB and refluxed at 60 ˚C in 50 mL acetone under argon atmosphere for 24 h. After cooling the reaction mixture to the room temperature, the solid potassium carbonate was removed using Whatman 40 filter paper. Afterward, acetone was evaporated using rota evaporator and the solid residue was dissolved in 50 mL ethyl acetate. Next, the crude product containing ethyl acetate layer was washed thrice with distilled water and passed over anhydrous Na_2SO_4. Finally, the crude product was purified through column chromatography (stationary phase: silica gel, Eluent: hexane and ethyl acetate). The final product appeared as white solid with 60-65% yield.

1,2-bis((6-bromohexyl)oxy)benzene (1a). Yield 65%; ^{1}H-NMR (400 MHz, $CDCl_3$): δ/ppm. 1.50-1.91 (m, -$OCH_2(CH_2)_4CH_2Br$, 16H), 3.42 (t, *J* = 6.8 Hz, -$OCH_2(CH_2)_2CH_2Br$, 4H), 4.0 (t, *J* =

6.5 Hz, -OCH_2(CH_2)$_2$$CH_2$Br, 4H), 6.88 (s, -$H_{Ar}$, 4H). HRMS (m/z): 435.0520, 437.0499 [$(M+H)^+$] (observed), 435.0529, 437.0508 [$(M+H)^+$] (calculated).

1,3-bis((6-bromohexyl)oxy)benzene (2a). Yield 64%; ^{1}H-NMR (400 MHz, $CDCl_3$): δ/ppm. 1.49-1.51 (m, -O(CH_2)$_2$(CH_2)$_2$(CH_2)$_2$Br, 8H), 1.77-1.80 (m, -O(CH_2)$_4$C$H_2$$CH_2$Br, 4H), 1.86-1.91 (m, -OCH_2CH_2(CH_2)$_4$Br, 4H), 3.42(t, J = 6.8 Hz, -O(CH_2)$_4$$CH_2CH_2$Br, 4H), 3.92-3.94 (t, J = 6.4 Hz, -OC$H_2$$CH_2$($CH_2$)$_4$Br, 4H), 6.44-6.49 (m, -$H_{Ar}$, 3H), 7.15 (t, J = 8.2 Hz, -H_{Ar}, 1H). HRMS (m/z): 435.0520, 437.0499 [$(M+H)^+$] (observed), 435.0529, 437.0508 [$(M+H)^+$] (calculated).

1,4-bis((6-bromohexyl)oxy)benzene (3a). Yield 65%; ^{1}H-NMR (400 MHz, $CDCl_3$): δ/ppm. 1.48-1.50 (m, -O(CH_2)$_2$(CH_2)$_2$(CH_2)$_2$Br, 8H), 1.75-1.78 (m, -O(CH_2)$_4$C$H_2$$CH_2$Br, 4H), 1.87-1.90 (m, -OCH_2CH_2(CH_2)$_4$Br, 4H), 3.42 (t, J = 6.8 Hz, -O(CH_2)$_4$$CH_2CH_2$Br, 4H), 3.91 (t, J = 6.4 Hz, -OC$H_2$$CH_2$($CH_2$)$_4$Br, 4H), 6.81 (s, -$H_{Ar}$, 4H). HRMS (m/z): 435.0559, 437.0533 [$(M+H)^+$] (observed), 435.0529, 437.0508 [$(M+H)^+$] (calculated).

Synthetic protocol of *N*,*N*,*N'*,*N'*-tetramethyl diaminohexyloxy benzenes (1b, 2b and 3b). $NHMe_2$ gas was collected into 20 mL chloroform solution of individual dibromohexyloxy benzene (**1a, 2a and 3a**) (1 g) in a sealed tube at 0 ˚C till the volume of the resulting solution was roughly doubled. Then, the reaction mixture was allowed to stir for 24 h at room temperature. Next, the reaction mixture was cooled and transferred into a RB. The solution was then kept in water bath to remove excess $NHMe_2$ followed by solvent evaporation by using rotary evaporator. Reaction mixture was then washed with 2 M aqueous KOH solution after dissolving it in $CHCl_3$. Finally, $CHCl_3$ layer was collected and evaporated to dryness to get a yellowish gummy liquid, **1b, 2b and 3b** with quantitative yield.

6,6'-(1,2-phenylenebis(oxy))bis(*N*,*N*-dimethylhexan-1-amine) (1b). ^{1}H-NMR (400 MHz, $CDCl_3$): δ/ppm. 1.36-1.40 (m, -O(CH_2)$_4$C$H_2$$CH_2$N($CH_3$)$_2$, 4H), 1.80-1.84 (m, -O($CH_2$)$_2$(C$H_2$)$_2$($CH_2$)$_2$N($CH_3$)$_2$, 8H), 1.79-1.83 (m, -O($CH_2$)$_4CH_2$$CH_2$N($CH_3$)$_2$, 4H), 2.20 (s, -$CH_2$N(C$H_3$)$_2$, 12H), 2.24-2.26 (m, -O(CH_2)$_2$$CH_2CH_2$N($CH_3$)$_2$, 4H), 3.99 (t, J = 6.6 Hz, -OC$H_2$$CH_2$($CH_2$)$_2$N($CH_3$)$_2$, 4H), 6.87 (s, -$H_{Ar}$, 4H). HRMS (m/z): 365.3154 [$(M+H)^+$] (observed), 365.3163 [$(M+H)^+$] (calculated).

6,6'-(1,3-phenylenebis(oxy))bis(*N*,*N*-dimethylhexan-1-amine) (2b). ^{1}H-NMR (400 MHz, $CDCl_3$): δ/ppm. 1.33-1.39 (m, -O(CH_2)$_4$C$H_2$$CH_2$N($CH_3$)$_2$, 4H), 1.44-1.84 (m, -O($CH_2$)$_2$(C$H_2$)$_2$($CH_2$)$_2$N($CH_3$)$_2$, 8H), 1.73-1.80 (m, -O($CH_2$)$_4CH_2$$CH_2$N($CH_3$)$_2$, 4H), 2.20 (s, -$CH_2$N(C$H_3$)$_2$, 12H), 2.24-2.26 (m, -O(CH_2)$_2$$CH_2CH_2$N($CH_3$)$_2$, 4H), 3.92 (t, J = 6.5 Hz, -OC$H_2$$CH_2$($CH_2$)$_2$N($CH_3$)$_2$, 4H), 6.44-6.48 (m, -$H_{Ar}$, 3H), 7.14 (t, J = 8.1 Hz, -H_{Ar}, 1H). HRMS (m/z): 365.3148 [$(M+H)^+$] (observed), 365.3163 [$(M+H)^+$] (calculated).

6,6'-(1,4-phenylenebis(oxy))bis(*N*,*N*-dimethylhexan-1-amine) (3b). ^{1}H-NMR (400 MHz, DMSO-d_6): δ/ppm. 1.23-1.31 (m, -O(CH_2)$_4$C$H_2$$CH_2$N($CH_3$)$_2$, 4H), 1.64-1.67 (m, -O($CH_2$)$_2$(C$H_2$)$_2$($CH_2$)$_2$N($CH_3$)$_2$, 8H), 1.64-1.67 (m, -O($CH_2$)$_4CH_2$$CH_2$N($CH_3$)$_2$, 4H), 2.08 (s, -$CH_2$$N^+$(C$H_3$)$_2$, 12H), 2.14-2.18 (m, -O(CH_2)$_2$$CH_2CH_2$N($CH_3$)$_2$, 4H), 3.89 (t, J = 6.5 Hz, -

$OCH_2CH_2(CH_2)_2N(CH_3)_2$, 4H), 6.81 (s, -$H_{Ar}$, 4H). HRMS (m/z): 365.3156 [$(M+H)^+$] (observed), 365.3163 [$(M+H)^+$] (calculated).

Synthetic protocol of ethyl ester bromide of phenylalanine (1c). Thionyl chloride (0.65 mL, 9 mmol) was added dropwise at 0-5 ˚C to 20 mL suspension of L-Phe (0.5 g, 3 mmol) in ethanol and the entire reaction mixture was refluxed for 12 h. Next, the excess ethanol and thionyl chloride were evaporated by rotary evaporator. The solid residue was washed with dry diethyl ether, resulted a white solid crude product. This crude white solid was dissolved in 10 mL of dichloromethane and potassium carbonate (1 g, 7.5 mmol) was added to the organic solution after dissolving it in 10 mL of distilled water. A solution of bromoacetyl bromide (0.4 mL, 4.5 mmol) in dichloromethane (10 mL) was then added dropwise to the reaction mixture at 5 °C for 1 h. The reaction mixture was stirred at room temperature for another 12 h. The aqueous solution was separated and the organic solution was washed with water and passed over the anhydrous Na_2SO_4 and concentrated to yield a white or yellowish white solid product with 80-94% yield.

Ethyl (2-bromoacetyl) phenylalaninate (1c). Yield 94%; ^{1}H-NMR (400 MHz, $CDCl_3$): δ/ppm. 1.24 (t, *J* = 7.1 Hz, -$COOCH_2CH_3$, 3H), 3.09-3.20 (m, -CH_2Ph, 2H), 3.85 (s, BrCH_2CONH-, 2H), 4.18 (q, *J* = 7.1 Hz, -$COOCH_2CH_3$, 2H), 4.79-4.83 (App quint., -$H_{(Phe)}$, 1H), 6.85-6.87 (br, -CON*H*, 1H), 7.12-7.28 (m, -H_{Ar}, 5H). HRMS (m/z): 314.0336, 316.0396 [$(M+H)^+$] (observed), 314.0386, 316.0366 [$(M+H)^+$] (calculated).

General synthesis procedure of isoamphipathic antibacterial molecules (IAMs **1-3).** Individually, ethyl ester bromide intermediates, **1c** (2.8 equiv.) were reacted with *N, N, N',N'*-tetramethyl diaminohexyloxy benzenes (**1b, 2b** and **3b**) (1 equiv.) in dry $CHCl_3$ (8-10 mL) at 65 ˚C. At the end of 24 h, reaction mixture was evaporated by using rotary-evaporator and the residue was dissolved in minimum amount of $CHCl_3$. The product was then precipitated by adding excess dry diethyl ether and the white residue was washed repeatedly with dry diethyl ether to remove the excess amount of activated ethyl ester bromides. The entire exercise resulted the generation of isoamphipathic antibacterial molecules (IAMs: **1-3**) with a quantitative yield. All the final molecules were characterized through ^{1}H-NMR, ^{13}C-NMR and HRMS.

IAM-1. ^{1}H-NMR (400 MHz, DMSO-d_6): δ/ppm.1.13 (t, *J* = 7.1 Hz, -$COOCH_2CH_3$, 6H), 1.26-1.31 (m, -$O(CH_2)_3CH_2CH_2CH_2N^+(CH_3)_2$-, 4H), 1.39-1.47 (m, -$O(CH_2)_2CH_2(CH_2)_2CH_2N^+(CH_3)_2$-, 4H), 1.63-1.75 (m, -$OCH_2CH_2(CH_2)_2CH_2CH_2N^+(CH_3)_2$-, 8H), 2.88-3.11 (m, -$CH_2$Ph, 4H and m, -$CH_2CH_2N^+(CH_3)_2$-, 12H), 3.35-3.39 (m, -$CH_2CH_2N^+(CH_3)_2$-, 4H), 3.94 (t, *J* = 6.6 Hz, -$COOCH_2CH_3$, 4H), 4.02-4.14 (m, -$OCH_2(CH_2)_5N^+(CH_3)_2$-, 4H), 4.02-4.14 (m, -$NHCOCH_2N^+(CH_3)_2$-, 4H), 4.52-4.60 (m, -$H_{(Phe)}$-, 2H), 6.85-6.96 (m, -H_{Ar}, 4H), 7.20-7.30 (m, -$H_{Ar\ (Phe)}$, 10H), 9.13 (d, *J* = 7.6 Hz -CON*H*, 1H). ^{13}C-NMR (100 MHz, DMSO-d_6): δ/ppm. 13.9, 21.7, 24.9, 25.3, 28.5, 51.2, 53.5, 60.9, 61.4, 61.5, 64.3, 68.1, 114.0, 120.9, 126.6, 128.2, 129.0, 136.6, 148.5, 163.2 and 170.6. HRMS (m/z): 416.2732 [$(M-2Br)/2^+$] (observed), 416.2670 [$(M-2Br)/2^+$] (calculated).

IAM-2. ^{1}H-NMR (400 MHz, DMSO-d$_6$): δ/ppm. 1.13 (t, *J* = 7.1 Hz, -COOCH$_2$C*H$_3$*, 6H), 1.26-1.31 (m, -O(CH$_2$)$_3$C*H$_2$*CH$_2$CH$_2$N$^+$(CH$_3$)$_2$-, 4H), 1.40-1.43 (m, -O(CH$_2$)$_2$C*H$_2$*(CH$_2$)$_2$CH$_2$N$^+$(CH$_3$)$_2$-, 4H), 1.61-1.72 (m, -OCH$_2$C*H$_2$*(CH$_2$)$_2$C*H$_2$*CH$_2$N$^+$(CH$_3$)$_2$-, 8H), 2.90-3.17 (m, -C*H$_2$*Ph, 4H and m, -CH$_2$CH$_2$N$^+$(C*H$_3$*)$_2$-, 12H), 3.34-3.36 (m, -CH$_2$C*H$_2$*N$^+$(CH$_3$)$_2$-, 4H), 3.92 (t, *J* = 6.2 Hz, -COOC*H$_2$*CH$_3$, 4H), 4.02-4.12 (m, -OC*H$_2$*(CH$_2$)$_5$N$^+$(CH$_3$)$_2$-, 4H), 4.02-4.12 (m, -NHCOC*H$_2$*N$^+$(CH$_3$)$_2$-, 4H), 4.55-4.61 (m, -*H*$_{(Phe)}$, 2H), 6.43-6.50 (s, -*H*$_{Ar}$, 3H), 7.15 (t, *J* = 8.2 Hz, -*H*$_{Ar}$, 1H), 7.20-7.31 (m, -*H*$_{Ar\ (Phe)}$, 10H), 9.07 (d, *J* = 7.5 Hz, -CON*H*, 1H). ^{13}C-NMR (100 MHz, DMSO-d$_6$): δ/ppm. 13.8, 21.6, 24.5, 25.3, 28.3, 51.3, 53.5, 61.0, 61.4, 67.1, 114.2, 120.8, 126.7, 128.3, 129.0, 136.5, 148.4, 159.8, 163.1 and 170.6. HRMS (m/z): 416.2737 [(M-2Br)/2$^+$] (observed), 416.2670 [(M-2Br)/2$^+$] (calculated).

IAM-3. ^{1}H-NMR (400 MHz, DMSO-d$_6$): δ/ppm. 1.13 (t, *J* = 7.1 Hz, -COOCH$_2$C*H$_3$*, 6H), 1.25-1.29 (m, -O(CH$_2$)$_3$C*H$_2$*CH$_2$CH$_2$N$^+$(CH$_3$)$_2$-, 4H), 1.39-1.44 (m, -O(CH$_2$)$_2$C*H$_2$*(CH$_2$)$_2$CH$_2$N$^+$(CH$_3$)$_2$-, 4H), 1.63-1.71 (m, -OCH$_2$C*H$_2$*(CH$_2$)$_2$C*H$_2$*CH$_2$N$^+$(CH$_3$)$_2$-, 8H), 2.90-3.35 (m, -C*H$_2$*Ph, 4H and m, -CH$_2$CH$_2$N$^+$(C*H$_3$*)$_2$-, 12H), 3.88 (t, *J* = 6.4 Hz, -COOC*H$_2$*CH$_3$, 4H), 3.98-4.10 (m, -OC*H$_2$*(CH$_2$)$_5$N$^+$(CH$_3$)$_2$-, 4H), 3.98-4.10 (m, -NHCOC*H$_2$*N$^+$(CH$_3$)$_2$-, 4H), 4.55-4.60 (m, -*H*$_{(Phe)}$, 2H), 6.82 (s, -*H*$_{Ar}$, 4H), 7.22-7.31 (m, -*H*$_{Ar\ (Phe)}$, 10H), 9.08 (d, *J* = 7.7 Hz, -CON*H*, 2H). ^{13}C-NMR (100 MHz, DMSO-d$_6$): δ/ppm. 13.9, 21.7, 25.0, 25.4, 28.4, 51.2, 53.5, 60.9, 61.3, 64.3, 67.6, 115.2, 126.6, 128.2, 129.0, 136.6, 152.6, 163.2 and170.6. HRMS (m/z): 416.2660 [(M-2Br)/2$^+$] (observed), 416.2670 [(M-2Br)/2$^+$] (calculated).

Antibacterial assay. [1,2]At first bacteria from the frozen stock (at -80 ˚C) were streaked either on nutrient broth (for Gram-positive bacteria) or MacConkey agar plate (for Gram-negative bacteria). The streaked plates were then incubated overnight at 37 ˚C for bacterial growth. A single bacterial colony was next inoculated for 6 h (midlog phase) in 3 mL of nutrient broth to produce about 10^8 to 10^9 CFU/mL cells depending upon the nature of the bacteria. The 6 h grown culture was diluted to ~10^5 CFU/mL which was then used for antibacterial assay determination. Compounds were 2-fold serially diluted in a 96-well plate from the starting concentration using sterile Millipore water. Afterward, 180 µL of ~10^5 CFU/mL bacterial solution was added in each well containing 20 µL aqueous solution of the test compound. The plates were then incubated for 16-18 h at 37 ˚C in shaking condition. The O.D at 600 nm was recorded by using TECAN (Infinite series, M200 PRO) plate reader. Each concentration was triplicate and the experiment was performed twice and the antibacterial activity (MIC) was evaluated based on visual turbidity.

Antibacterial activity upon human blood plasma and serum pre-incubation. [2] Human blood was centrifuged at 3500 rpm for 5 min and the plasma was collected carefully from the supernatant. Similarly, human blood was collected into BD Vacutainer® Serum Tubes and further centrifuged at 3500 rpm to obtain human blood serum. Next, 500 µL of 512 µg/mL (515.6 µM) solution of IAM-**1** in 1×PBS was mixed with equal volume of blood plasma/serum and the individual mixtures were incubated for different time points (1 h, 2 h, and 3 h) at 37 ˚C. At the end of specific incubation time, each mixture was subjected for 2 fold serial

dilutions and MIC was conducted with them by following the aforementioned protocol against MRSA.

Hemolytic activity.[2,3] Aqueous solution of individual compounds (IAMs: **1-3**) were serially diluted by two fold in triplicate in a 96-well plate. Freshly collected human blood (heparinized) was then centrifuged down and supernatant was thrown away to collect the human red blood cells (hRBCs). Later, collected hRBCs (5 vol %) were slowly suspended using 1×PBS (pH = 7.4). Next, 150µL of this suspension was added to the wells of 96 well plates containing 50 µL compound's solution and plate was allowed to incubate at 37 ˚C for 1 h. Then centrifugation at 3500 rpm for 5 min was performed and the supernatant (100 µL) was then transferred to another 96-well plate for recording the absorbance at 540 nm by using Tecan Infinite M200 PRO microplate reader. In this study, same volume of 1×PBS without compound served as a negative control whereas same volume of Triton X-100 (1 vol% solution in 1×PBS) was used as a positive control. The percentage of hemolysis was determined by using the following formula: $(A_{treat} – A_{nontret}) / (A_{TX\text{-}treat} – A_{nontret}) \times 100$, where A_{treat} corresponds to the absorbance of the compound-treated well, $A_{nontret}$ stands for the absorbance of the negative controls (without compound), and $A_{TX\text{-}treat}$isthe absorbance of the triton-X-100 treated well. Each concentration had triplicate values and the HC_{50} was determined by considering the average of triplicate O.D.

Co-culture study

Co-culture with MRSA and human erythrocytes.[4] MRSA and human erythrocytes were mixed with each other in 1×PBS in such way so that their effective concentration remained as ~10^7 CFU/mL and ~10^8 hRBCs/mL. Next, 180 µL of the co-culture suspension was treated individually with 20 µL of IAM **1-3** at 2560 µg/mL (2578 µM) [working concentration: 256 µg/mL (257.8 µM)]. After 15 min incubation at 37 ˚C in a stationary condition, 20 µL of the suspension was withdrawn and 10-fold serially diluted followed by spot plating each dilutions on nutrient agar plates for counting viable MRSA cells. Finally, at the end of 24 h incubation, the viable bacterial colonies were counted and the results were expressed in percentage with respect to the untreated MRSA count in co-culture condition. On the other hand, the reaming volume of MRSA and hRBC suspension was centrifuged at 3500 rpm for 5 min and the absorbance of the supernatant (100 µL) was recorded at 540 nm by using Tecan Infinite M200 PRO microplate reader. In this study, same volume (20 µL) of triton-X (1 vol% solution in 1×PBS) instead of test compound was used as a positive control for determination of hRBC lysis. The percentage of hemolysis was determined by using the following formula: $(A_{tret} – A_{nontret}) / (A_{TX\text{-}tret} – A_{nontret}) \times 100$, where A_{tret} corresponds to the absorbance of hRBCs in co-culture condition upon compound treatment , $A_{nontret}$ stands for the absorbance of hRBCs in co-culture condition without compound treatment, and $A_{TX\text{-}tret}$ is the absorbance of hRBCs in co-culture condition upon triton-X treatment.

Co-culture with MRSA and RAW cells.[5]RAW 264.7 cells (~10^5 cells/well) were seeded onto the wells of a 96-well plate in DMEM media (supplemented with 10% FBS and 5% penicillin-

streptomycin) at 37 ˚C with 5 % CO_2 atmosphere for 12 h. Afterward, cell culture medium was discarded and cells were treated with 100 µL of freshly prepared MRSA (~ 10^5 CFU/mL) supplemented with 256 µg/mL (257.8 µM) compound solution (IAM-**1** or IAM-**2** or IAM-**3**) in antibiotic free DMEM media with 10% FBS. Two controls were included in the study, in one case the MRSA cells were left untreated with compound in presence of RAW cells and in other case, RAW cells were left untreated with both compound as well as MRSA where same volume of antibiotic-free DMEM (supplemented with 10% FBS) was added. The plate was then incubated for 3 h at 37 ^{0}C under 5% CO_2 atmosphere. To determine the bacterial cell viability, 20 µL of the suspension was withdrawn and 10-fold serially diluted followed by spot plating each dilution on nutrient agar plates for counting viable MRSA cells. Finally, at the end of 24 h incubation, the viable bacterial colonies were counted and the results were expressed in percentage with respect to the untreated MRSA count in co-culture condition. On the other side, RAW cells viability was also determined by performing Alamar blue assay (chapter 2, section 2.4.12) with the reaming supernatant and results were represented in terms of percentage by considering 100% cell viability in case of RAW cells devoid of MRSA and compound treatment.

Membrane fluidity study.[6,7] 0.5 mM of DPPG : DPPE (88 : 12) and 0.5 mM of DPPC lipids along with laurdan dye (5 mM) were dissolved in minimal volume of analytical grade chloroform in a 10 mL RB. Next, a thin layer films with the lipids were made within the walls of RB by applying argon flow. Further, the thin films were dried under vacuum for 1 h. Then, 10 mL of 1×PBS (pH = 7.4) was added on the lipid films and incubated for 12 h at 4-8 ˚C for hydration. Further, 10 freeze-thaw cycles (from 70 ˚C to 4 ˚C with intermittent vortexing) were executed with the hydrated films. These multilamellar vesicles were finally sonicated at 70 ˚C for 15 min to obtain unilamellar vesicles. The freshly prepared liposome solution (2 mL) was taken into 3 mL fluorescence cuvette and the emission intensity was measured at 440 nm upon excitation at 350 nm. Similarly, the emission intensity was measured upon IAMs **1-3** treatment at 256 µg/mL (257.8 µM) in case of both bacterial and mammalian membrane mimicking liposomes.

Membrane leakage study.[6,7] To investigate dye leakage upon IAM **1-3** treatment through fluorescence spectroscopy, vesicles entrapped with 30 mM 5(6)-Carboxyfluorescein (CF) dye was prepared in 1×PBS buffer (pH = 7.4). In high concentrations (30 mM), CF does not emit due to self-quenching and displays significant emission at diluted condition of 3 mM concentration. Bacterial membrane mimicking vesicles were prepared using 0.5 mM DPPG and DPPE lipid (88 : 12) in CF solution (30 mM) in 1×PBS buffer by following the previously mentioned protocol. Mammalian membrane mimicking vesicles were also prepared in the similar way using 0.5 mM DPPC lipid. However, herein, each freeze-thaw cycle was accompanied with sonication for 30 s. Further, vesicle untapped dye was removed from the solution using size exclusion chromatography. Sephadex G-50 was used as the stationary phase and 1×PBS was used as the eluent. Finally, 200 µL of vesicles were equilibrated with 1.8 mL 1×PBS and emission intensity was recorded at 517 nm using an

excitation at 495 nm. In control case, instead of test compound solution, water was added at 50 s and fluorescence intensity was measured upto 200 s. Likewise, fluorescence intensity was recorded upon treating bacterial and mammalian membrane mimicking vesicles individually with IAM **1-3** [160 μg/mL (161 μM)] at 50 s followed by addition of 1% triton-X at 200 s. The fluorescence intensity obtained after triton-X treatment in case of control was used for normalization. Further, rate constant of dye leakage was calculated by fitting each emission spectrum from t = 50 s (after addition of IAM) to t = 200 s (before addition of triton-X) using exponential rise equation ($y = y_0 + A_1e^{-x/t_1}$). The rate constant was considered ask = $1/t_1$.

Water-membrane partition experiments

Liposomes were prepared by extrusion, as previously described, with lipid compositions: POPE/POPG (7:3, mol/mol) and POPC/Cholesterol (1:1, mol/mol), as mimics of bacterial and eukaryotic membranes, respectively. The lipid film was hydrated with a 10 mM phosphate buffer (pH=7.4), prepared by using ultrapure water and containing 140 mM NaCl and 0.1 mM EDTA (buffer A henceforth). Liposomes were separated from unencapsulated dye by gel filtration on a 40 cm Sephadex G-50 column. The final lipid concentration was determined according to the Stewart phospholipid assay.[8]

A fixed IAM concentration (10 μM) was titrated with increasing concentrations of liposomes. After each addition, the fluorescence spectra were recorded repeatedly until no further changes were observed (about 5-10 minutes). Control experiments to check for possible effects of sample turbidity on the fluorescence signal were performed by repeating the same titration with tryptophan.

The fraction of IAM associated to membranes (f_b) was calculated from the fluorescence intensity (F, at the wavelength of 318 nm), according to the following equation:

$$f_b = \frac{(F - F_w)}{(F_b - F_w)} \quad (1)$$

where F_b and F_w refer to the fluorescence signal of bound and free IAM, respectively. F_b was determined by extrapolating the titration with the following equation:

$$F = F_W + (F_b - F_W)\frac{K_p \cdot \frac{[L]}{[W]}}{1 + K_p \cdot \frac{[L]}{[W]}} \quad (2)$$

where K_p represents the lipid/water partition constant, $[L]$ is the molar concentration of lipids in the sample and $[W]$ is the molar concentration of pure water at 25 °C (55.5 M). In Equation 2, the lipid concentration dependence of the fraction of bound IAM was described by the following equation, corresponding to an ideal partition equilibrium model.[8,9]

$$f_b = \frac{K_p \cdot \frac{[L]}{[W]}}{1 + K_p \cdot \frac{[L]}{[W]}} \quad (3)$$

The partition curves of the POPC/Cholesterol neutral liposomes were too far from the plateau to allow extrapolation. Therefore, the value of the bound state fluorescence F_b determined for the POPE/POPG liposomes was used for the POPC/Cholesterol vesicles, too, assuming that the membrane composition does not affect significantly the fluorescence in the bound state. In principle, the turbidity of the liposome solution might artifactually cause variations in the fluorescence signal. However, negative control experiments with tyrosine (which does not bind to vesicles) showed no significant changes in the emission spectra, demonstrating the absence of any relevant scattering-related artefacts.

Molecular dynamics simulations

The force field parameters for IAM compounds were obtained by starting from the ATB server.[10] The charges were slightly modified to preserve the symmetry of the molecules and in analogy with those of analogous groups in the GROMOS 54A7 force field.[11] The hydrophobic chains in the compounds were modeled with the Berger parameters used for lipids (see below).

In simulations of the IAM molecules in water, a single copy of the compound was placed at the center of a box (44 nm^3) and hydrated with approximately 1400 pre-equilibrated simple point charge (SPC) water molecules. 6 Cl^- and 4 Na^+ ions were added to ensure electroneutrality and a ionic strength roughly corresponding to 0.150 M. MD simulations were carried out with the GROMACS 2020.6 software package.[12] Each system was energy-minimized and then equilibrated during a 100 ps MD, where the positions of the IAM atoms were restrained. Production simulations were performed at least in triplicate, for a total amount of almost 12 µs of simulation time at a temperature of 300 K, with the same parameters and settings used in the membrane simulations (see below).

The most representative structures of each simulation were defined by cluster analysis conducted with GROMACS (cluster tool), using a 0.25 nm root mean square deviation cutoff.

MD simulations of IAM-1 in presence of membrane lipids were performed with the “minimum bias” approach, which minimizes the effect of the initial configuration on the final results. In this method, the simulation is started from a random mixture of the membrane-active molecule, lipids and water, and the bilayer forms spontaneously, usually in 50–100 ns. During this self-assembly process, the system is very fluid, particularly in the first stages of the simulation, thus ensuring that the bioactive molecule can sample different environments in a relatively short time, and, as a consequence, it can find its minimum free energy configuration. We demonstrated that the simulative results obtained with this approach are consistent with the depth of membrane insertion and the orientation

determined experimentally by fluorescence, ATR-FTIR and solid-state NMR spectroscopies.[13-15] Membranes of POPE/POPG (90:38) were used to parallel the conditions of the experimental studies on lipid vesicles and to mimic bacterial membranes. Briefly, a single copy of the compound was placed at the center of a 9 × 9 × 9 nm box. 128 lipid molecules and 7500 water molecules were randomly added into the box. 38 Na^+ atoms were introduced in replacement of water molecules, as counterions of the negative charges on the lipids, and 2 Cl^- atoms as counterions of the +2 charge on the compounds. MD simulations were carried out with the GROMACS 2020.6 software package. The parameters for the lipids were taken from the literature.[16] Temperature was controlled using a velocity-rescaling thermostat.[17] Pressure coupling was applied using the Parrinello-Rahman barostat, with a time constant of 1.0 ps and a reference pressure of 1 bar.[18] All bond lengths were constrained with the LINCS algorithm.[19] Short-range electrostatic interactions were cut-off at 1.4 nm and long-range electrostatic interactions were calculated using the particle mesh Ewald (PME) algorithm.[20] Simulations were run with a 2 fs time step. Each system was energy minimized and then equilibrated using a 100 ps MD, where the positions of the IAM atoms were restrained. At first, simulations were performed for 200 ns, at a temperature of 310 K, with anisotropic pressure coupling. In cases where this time was not sufficient to attain a defect-free bilayer, the system was annealed by cycling the temperature between 310 K and 375 K, for further 120 ns, followed by a production run with semi-isotropic pressure coupling. Analyses were conducted on the last 20 ns of the simulations. The density profiles along the bilayer normal were determined by means of the “gmx density” tool in GROMACS.

Molecular graphics images were produced using the UCSF Chimera package from the Resource for Biocomputing, Visualization, and Informatics at the University of California, San Francisco (supported by NIH P41 RR-01081).[21]

Membrane active mechanism of action. [1-3]

Cytoplasmic Membrane Depolarization Assay. Midlog phase (working concentration: ~10^8 CFU/mL) MRSA cells were collected separately (centrifugation at 3500 rpm for 5 min), and washed with 1:1 ratio of 5 mM glucose and HEPES buffer (pH = 7.4). Next, the bacterial plate was resuspended in 1:1:1 ratio of 5 mM HEPES buffer, 100 mM KCl solution supplemented with 0.2 mM EDTA and 5 mM glucose. For this study EDTA was used to allow the dye uptake by permeabilizing outer membrane of *A. baumannii*. This study was performed in a Corning 96 black well plate with clear bottom containing 2 µM of 3,3′-dipropylthiadicarbocyanine iodide [$DiSC_3(5)$] and 190 µL of bacterial suspension. After 60 min incubation of the plate, fluorescence intensity was measured at 622 nm excitation wavelength and 670 nm emission wavelength for 4 min. After that, 10 µL of IAM-**1** (at working concentration of 16 µg/mL and 32 µg/mL) was mixed with the suspension of bacteria and dye of each well. Same volume of water without compound was used as the control for this experiment. Increment in

fluorescence intensity was measured for another 25 min using Tecan Infinite M200 PRO microplate reader.

Outer Membrane (OM) Permeabilization Assay. Midlog phase *E. coli* cells were independently harvested, washed with 1:1 mixture of 5 mM HEPES buffer and glucose and resuspended with the same. The working concentration of midlog phase and stationary phase bacteria were ~10^8 CFU/mL. This study was performed in a Corning 96-black well plate with clear bottom containing 10 µM of *N*-Phenyl naphthylamine (NPN) dye and 190 µL of bacterial suspension. Then, the fluorescence was monitored for first 4 min at excitation wavelength of 350 nm and emission wavelength of 420 nm. After that, bacterial suspension with dye at each well was treated with 10 µL of test compound, IAM-**1** at working concentrations of 16 µg/mL and 32 µg/mL. The same volume of water without a compound was used as the control for this experiment. An increase in fluorescence intensity was monitored for another 25 min with Tecan Infinite M200 PRO microplate reader.

Bacterial live/dead assay.[1]IAM-**1** [16 µg/mL (16 µM)] was added to 1 mL of ~10^8 CFU/mL midlog phase MRSA suspension in normal saline and was incubated at 37 ˚C. After 6 h of incubation, bacterial suspension was centrifuged to remove the compound completely. Then, the bacterial palate was resuspended with normal saline followed by the addition of 5 µL of Syto-9 (3 µM) and PI (15 µM) mixture and incubated for half an hour. Further, the dye containing bacterial suspension was centrifuged to remove the excess unbound dye and the palate was resuspended with 50 µL of normal saline. Finally, 5 µL of bacterial suspension was taken into a glass slide and processed for confocal microscopy at 63 X resolution.

Cytotoxicity assay.[2]

Alamar blue Assay. Cytotoxicity of IAMs: **1-3** was examined against Raw 264.7 cell line by Alamar blue assay. Briefly, cells (~10^4 cells/well) were seeded onto the wells of a 96-well plate in DMEM media supplemented with 10% fetal bovine serum (FBS) and 5% penicillin-streptomycin. Then 100 µL of serially diluted compound solution in DMEM media was added to the each well of the plates containing the cells. The same volume of media (untreated cells) and the cells treated with 0.1% (v/v) Triton-X solution was taken as positive and negative control respectively. The plates were then kept for incubation at 37 ˚C for 24 h maintaining 5% CO_2 atmosphere. Afterward, 10 µL of 10 x Alamarblue solution was added to each well followed by 4 h of further incubation at the same condition. Then, the absorbance was recorded at 570 nm wavelength and 600 nm wavelength was used as the reference. The percentage of cell viability was calculated using the following equation: cell viability (%) = $(A_c - A_t)/(A_0 - A_t) \times 100$, where A_c indicates the absorbance for cells treated with compound, A_t is the absorbance for the cells treated with 0.1% (v/v) Triton-X and A_0 is the absorbance of the untreated cells, all at 570 nm. Each concentration had triplicate values, and the average of triplicate absorbance values was plotted against concentration followed by fitting with a sigmoidal plot.

Fluorescence Microscopy. ~10^4 cells (HEK 293) were seeded into the individual wells of a 96-well plate. Then 100 µL of 256 µg/mL of IAMs: **1-3** was added over the seeded cells. 0.1% Triton-X treated and untreated cells were considered as positive and negative controls respectively. After single time washing with 1×PBS the untreated and treated cells were then stained with 50 µL of 1:1 calcein-AM (2 µM) and propidium iodide (PI) (4.5 µM) for 15 min under 5% CO_2 atmosphere at 37 ˚C. Finally, the excess dyes were removed by washing the cells with 1×PBS, and images were captured at 40×objective with the help of a Leica DM2500 fluorescence microscope. During imaging, a band-pass filter for calcein-AM (at 500−550 nm) and a long-pass filter for PI (at 590−800 nm) were used.

Bactericidal kinetics.[2] A single colony of MRSA and *E. coli* was inoculated separately in nutrient broth for 6 h at 37 ˚C to produce 10^8 CFU/mL to 10^9 CFU/mL cells. Next, this midlog phase bacterial solution was further diluted to ~5× 10^5 CFU/mL and 180 µL of this diluted bacterial solution in Mueller Hinton broth was added to the 20 µL aqueous solution of test compound**,** IAM-**1** with the concentration of 16 µg/mL (16.1 µM), 32 µg/mL (32.2 µM) for MRSA and 32 µg/mL (32.2 µM) and 64 µg/mL (64.4 µM) for *E. coli*. The same volume of autoclaved water without the test compound was used as a control. Afterward, 20 µL of aliquots from the individual mixture of bacteria and compound were serially diluted by 10-fold in sterile saline at different time points (0 h, 1 h, 2 h, 4 h, 6 h, and 12 h). Then, spot plating on agar plates was executed with 20 µL solution from each dilution and allowed to incubate for 24 h at 37 ˚C. Finally, the number of bacterial colonies was counted and results were presented in logarithmic scale, i.e., Log (CFU/mL) vs time.

Activity against stationary and persister cells.[2,3]A midlog phase (6 h grown culture) MRSA and *E. coli* culture was diluted to 1:1000 ratio in nutrient broth and incubated at 37 ˚C for 16 h in shaking condition to achieve stationary phase cells. Later on, the bacterial suspension was centrifuged (9000 rpm, 2 min) and resuspended in 1xPBS. On the other hand, the persister cells were generated from the stationary phase cells upon specific antibiotic exposure for 3 h. 1 mL of stationary phase culture was treated with 100 µg/mL (for *S. aureus*) and 300 µg/mL (for *E. coli*) of ampicillin sodium for 3 h at 37 ˚C. Next, the bacteria were centrifuged, washed 3-4 times, and resuspended in 1xPBS to remove the traces of the antibiotic. Finally, 180 µL of the stationary and persister phase bacteria (~5× 10^5 CFU/mL) was added to 20 µL of IAM-**1** solution with the concentrations of 16 µg/mL (16.1 µM), 32 µg/mL (32.2 µM) for stationary phase MRSA and persister phase *S. aureus* and 32 µg/mL (32.2 µM), 64 µg/mL (64.4 µM) for both stationary and persister phase *E. coli*. Similarly, for vancomycin (stationary phase MRSA and persister phase *S. aureus*) and colistin (for both stationary and persister phase *E. coli*) antibiotic concentrations were 64 µg/mL (64.4 µM) and 32 µg/mL (27.7 µM) and same volume of water without compound was considered as an untreated control. After 6 h time, 20 µL aliquots from that solution were serially diluted 10-fold in sterile saline. Then 20 µL solution from each dilution was spot plated on MacConkey agar plates and after 24 h of incubation at 37 ˚C, the number of bacterial colonies was

counted. The results were presented in a bar plot in logarithmic scale, i.e. Log (CFU/mL) at 6 h time point.

Biofilm disruption assay. [2,3]

Crystal Violet Staining. Biofilm disruption study was conducted over coverslips of diameter 13 mm. First, 6 h grown culture of MRSA ATCC33591 (midlog phase) was diluted to 10^5 CFU/mL in nutrient broth, supplemented with 1% glucose and 1% NaCl. Then this diluted bacterial suspension (2 mL/well) was added to wells containing the sterilized coverslips and the plate was allowed to incubate at static condition at 37 ˚C for 24 h. Then, after removal of media, biofilm containing coverslips were cautiously washed with 1×PBS (pH = 7.4) to remove the planktonic bacteria and coverslips were then placed into the well of a new 6-well plate. Afterward, two solutions (2 mL) of IAM-**1** with two different concentrations [64 μg/mL (64.4 μM) and 128 μg/mL (128.9 μM)] were added to the wells containing biofilm coated coverslip incubated for 24 h. As an untreated control, 2 mL of fresh media without compound was added to the well. For this study, vancomycin at 64 μg/mL (44.2 μM) was used as the antibiotic control. After 24 h, 1×PBS was used to wash the planktonic cells from the coverslips. Later on, all the compound treated and untreated coverslips were carefully positioned into another 6-well plate. To visualize biofilm disruption, those coverslips were incubated with 1 mL of 0.1% of crystal violet (CV) dye in the 6-well plate and allowed to incubate for 10 min. After washing with 1× PBS, the crystal violet associated with the biofilm containing coverslip was dissolved in 95% ethanol and absorbance was recorded at 520 nm. The amount of biomass left on the coverslips was indicated by the absorbance of CV dye.

Cell Viability of Biofilm Embedded Bacteria. In this case, an aforementioned protocol was followed for biofilm growth and treatment with IAM-**1** and antibiotics. After placing the washed coverslips containing biofilm into a fresh well plate, 2 mL of trypsin-EDTA solution diluted in saline (1:4 ratios) was added and allowed to incubate for 10-15 min with shaking. 20 µL of the bacterial suspension was then 10-fold serially diluted and each dilution was spot plated on nutrient agar plates. The spotted plates were then incubated for 24 h. At the end of incubation, the viable bacterial colonies were counted and the results were expressed as Log (CFU/mL).

Cell Viability of Biofilm Disseminated Bacteria. To quantify the viability of biofilm disseminated bacterial cells, 20 µL of dispersed cell suspension present in the biofilm growing media was serially 10 fold diluted in 0.9% saline and 20 µL of the diluted solutions was spot plated on nutrient agar plate and allowed to incubate for 18 h at 37 ˚C. Afterward, the viable bacterial colonies were counted.

Confocal Laser-Scanning Microscope (CLSM) of Biofilms. Like the previous studies, the optimized molecule, IAM-**1** had been used for this experiment. The treated and untreated coverslips (previously described in biofilm disruption assay section) were placed on glass slides after washing with 1×PBS. The biofilms staining was performed with 5 µL of Syto-9 (60

μM) and PI (15 μM) dye mixture and images were captured with the help of a Zeiss 510 Meta confocal laser-scanning microscope. Image J was used to process the images. Further, the percentage of live and dead cell ratio was calculated through Image J using the following formula.

% live cells = [(No of cells stained with green colour-No of cells stained with red colour) x100]/No of cells stained with green colour

% Dead cells = (100 - % of live cells)

Dermal Toxicity.[2] The study was executed by following OECD guidelines (OECD 425). Briefly, three groups of mice (n = 4) were used for this experiment. First, the mice were anesthetized by using 150 μL of xylazine-ketamine cocktail through intraperitoneal injection. Then, fur from the dorsal area (~1/10th of total body surface) of mice were removed carefully without any skin injury. Afterward, IAM-**1** with 200 mg/kg dosage in saline was applied to the shaved portion of one group of mice. Other group of mice was also treated with saline without any compound. The compound treated group of mice was continuously monitored for first 2 h from the initial experimental time point. Later on, both group of mice was monitored for another 14 days. On 7th day of post treatment, one group of mice treated with IAM-1 were sacrificed through cervical dislocation and the dermal tissues were isolated in 10% neutral buffer formalin (NBF) in order to analyse histopathological changes through hematoxylin and eosin staining. Similarly, other group of mice treated with IAM-**1** were also sacrificed on 14th day and their dermal tissues were subjected to histopathological analysis. This part of the experiment was performed at Rohana Veterinary Diagnostic laboratory (Bangalore, Karnataka, India).

***In-vivo* antibacterial assay.** 6-8 week BALB/C CD-1 female mice with average body weight of 22 g were assigned as 4 number in each treatment group. First, the mice were anesthetized by using 150 μL of xylazine-ketamine cocktail through intraperitoneal injection. Then, fur from the dorsal area (~1/10th of total body surface) of mice was removed carefully without any skin injury. Next, a wound was created on the dorsal area using scalpel. Then, the wound site was infected with 20 μL of 10^7 CFU midlog phase (6 h grown) MRSA ATCC 33591 in 1×PBS. The treatment with IAM-**1** (40 μL in 1×PBs, 80 mg/kg, once in a day) and vancomycin (40 μL in 1×PBS, 50 mg/kg, once in a day) was started after 4 h post infection upon topical application. The treatment was continued for next 5 consecutive days. In case of control group of mice, 40 μL of 1×PBS was applied topically for total 5 days. Finally, at 6th day, all the group of mice were euthanized through cervical dislocation and the infected skin tissue were collected aseptically in 10 mL saline. In general, the weight of the mice dermal tissue was in the range of 0.16-0.46 g. Next, these tissues were homogenised in 10 mL saline and the number of MRSA in this infected tissue was tittered. The bacterial count was expressed in Log CFU/gm.

Supplementary Figures

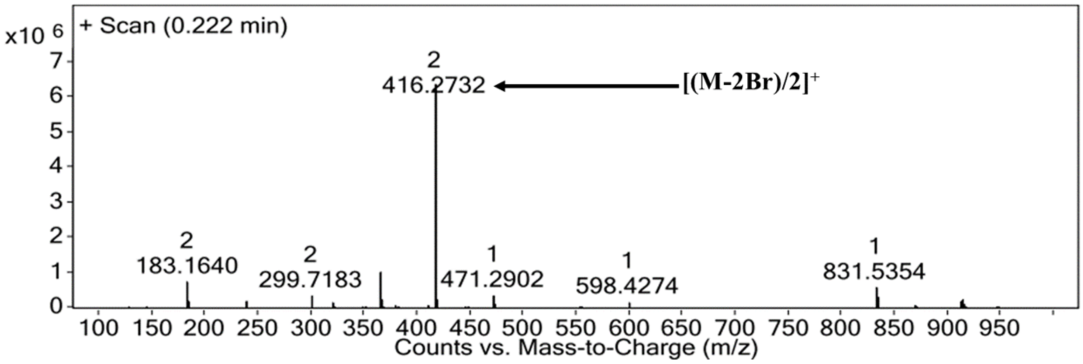

Figure S1. HRMS of **IAM-1**.

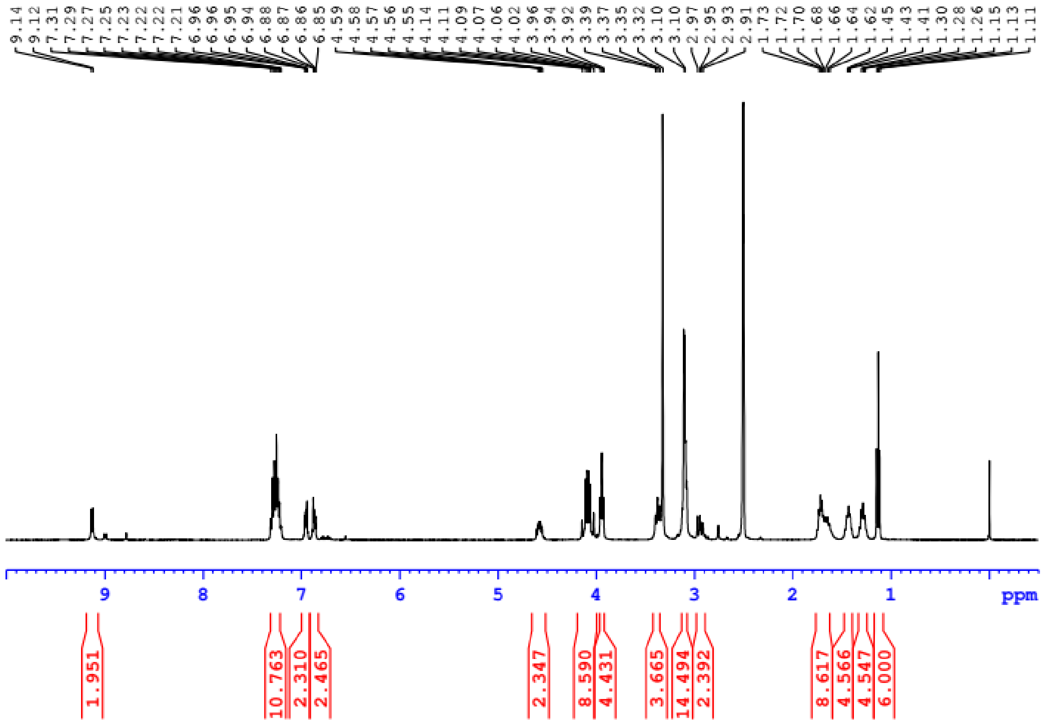

Figure S2. ^{1}H-NMR of IAM-**1**. The NMR was taken in DMSO-d6 and the solvent peak was calibrated at the δ value of 2.5 ppm.

.

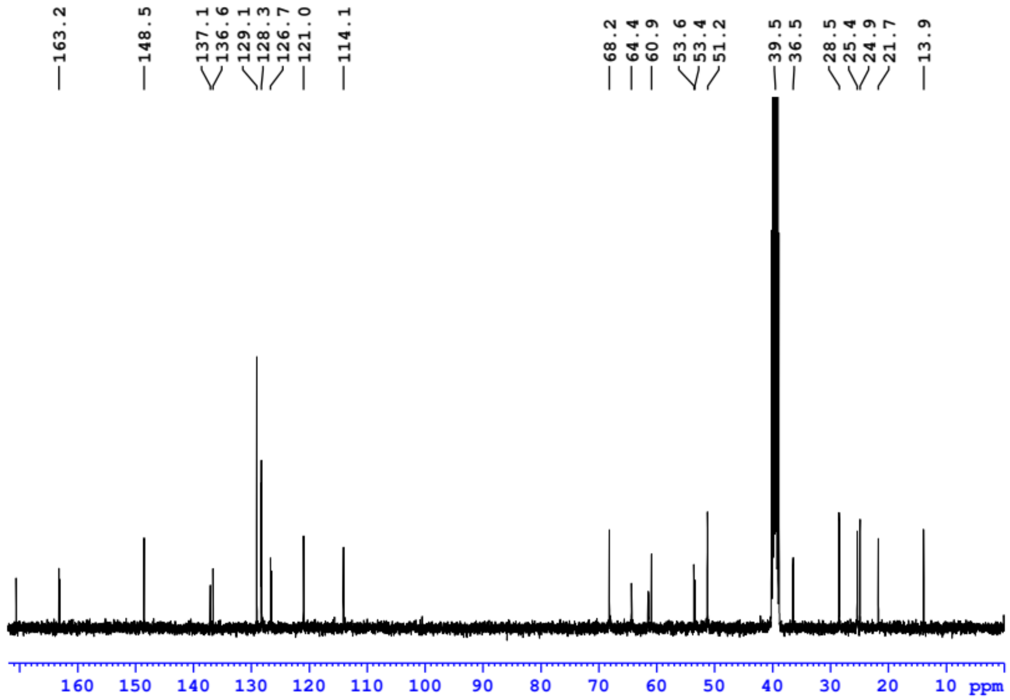

Figure S3. ^{13}C-NMR of **IAM-1**. The NMR was taken in DMSO-d_6 and the solvent peak was calibrated at the δ value of 39.52 ppm.

.

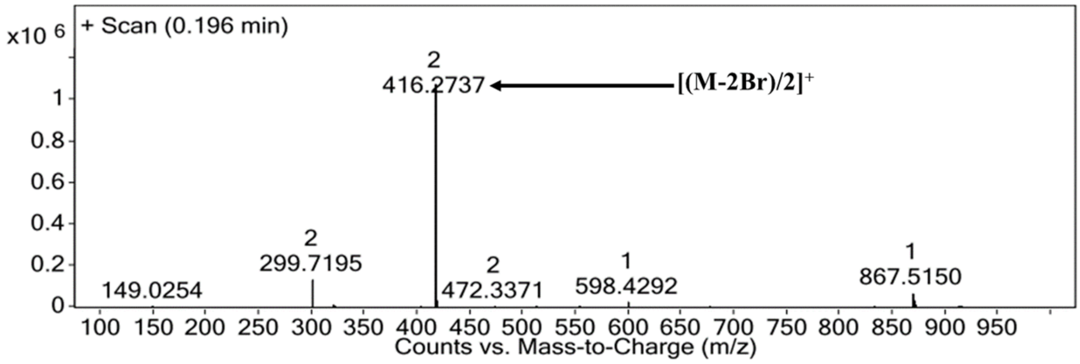

Figure S4. HRMS of **IAM-2**.

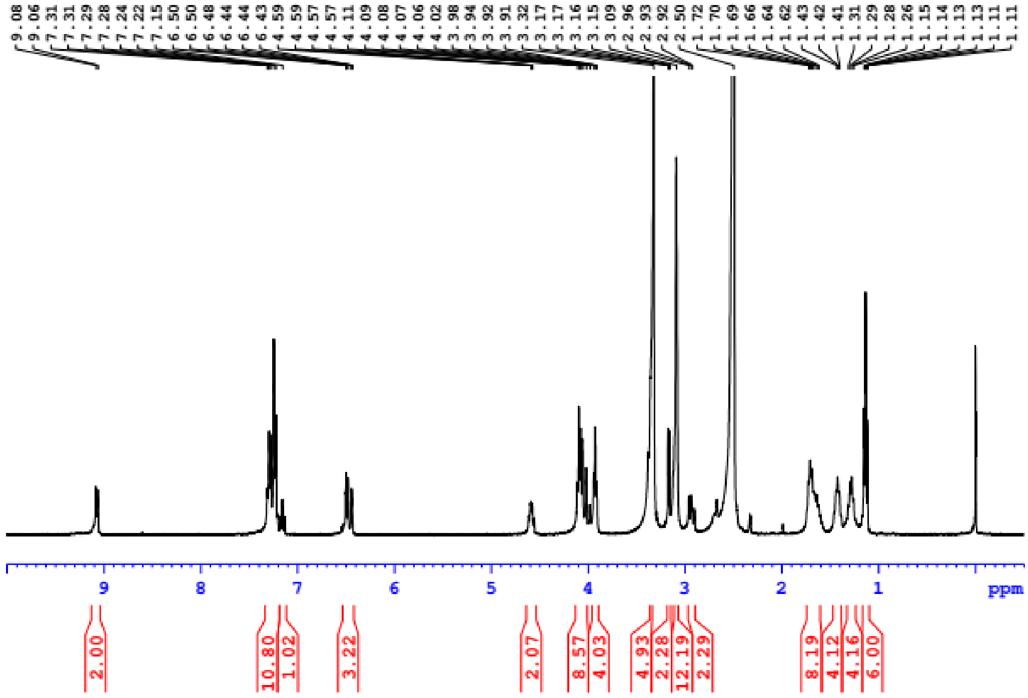

Figure S5. 1H-NMR of IAM-**2**. The NMR was taken in DMSO-d6 and the solvent peak was calibrated at the δ value of 2.5 ppm.

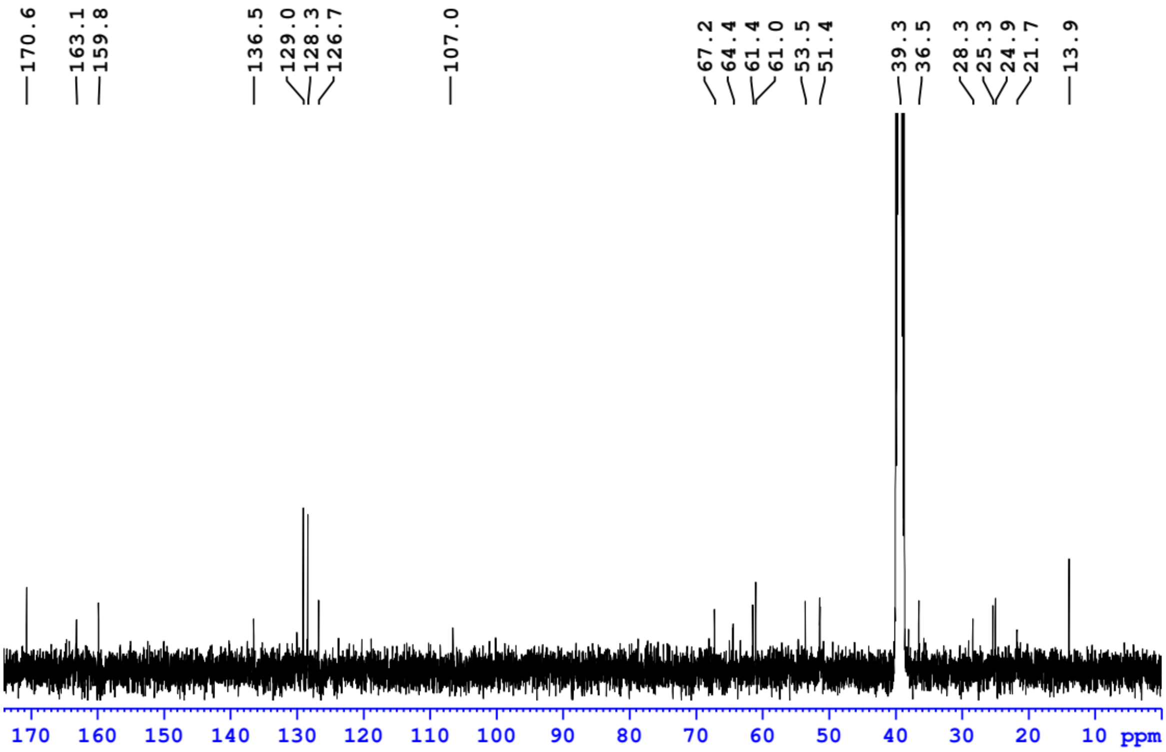

Figure S6. ^{13}C-NMR of **IAM-2**. The NMR was taken in DMSO-d_6 and the solvent peak was calibrated at the δ value of 39.52 ppm.

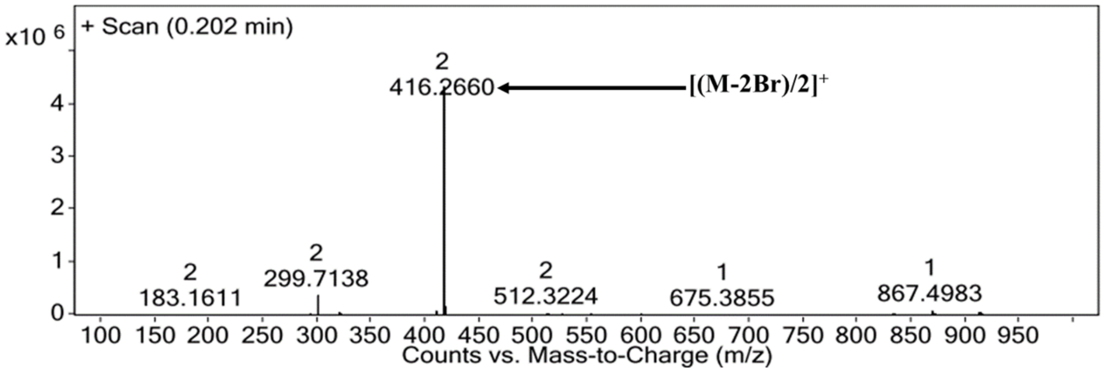

Figure S7. HRMS of **IAM-3**.

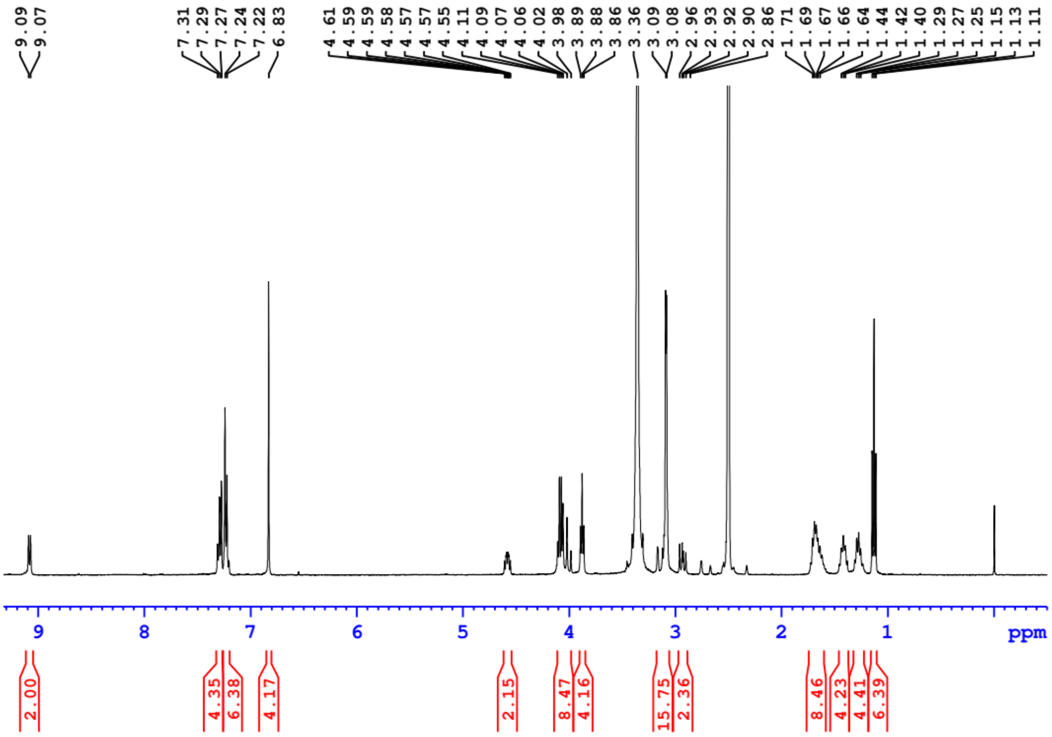

Figure S8. ^{1}H-NMR of IAM-**3**. The NMR was taken in DMSO-d6 and the solvent peak was calibrated at the δ value of 2.5 ppm.

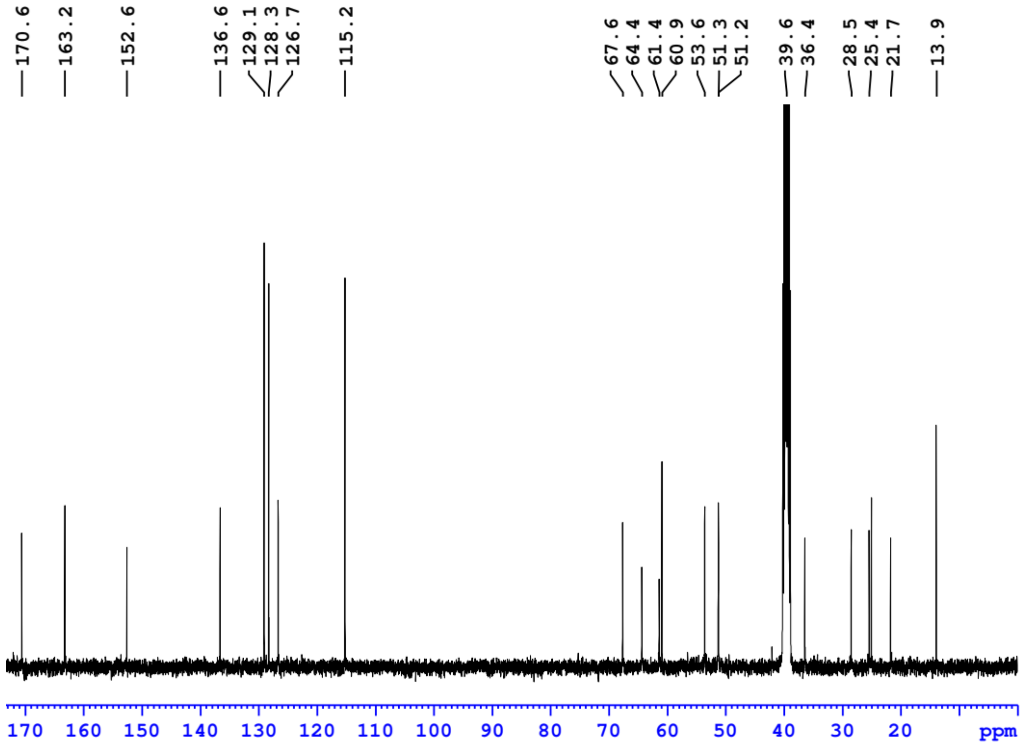

Figure S9. ^{13}C-NMR of **IAM-3**. The NMR was taken in DMSO-d_6 and the solvent peak was calibrated at the δ value of 39.52 ppm.

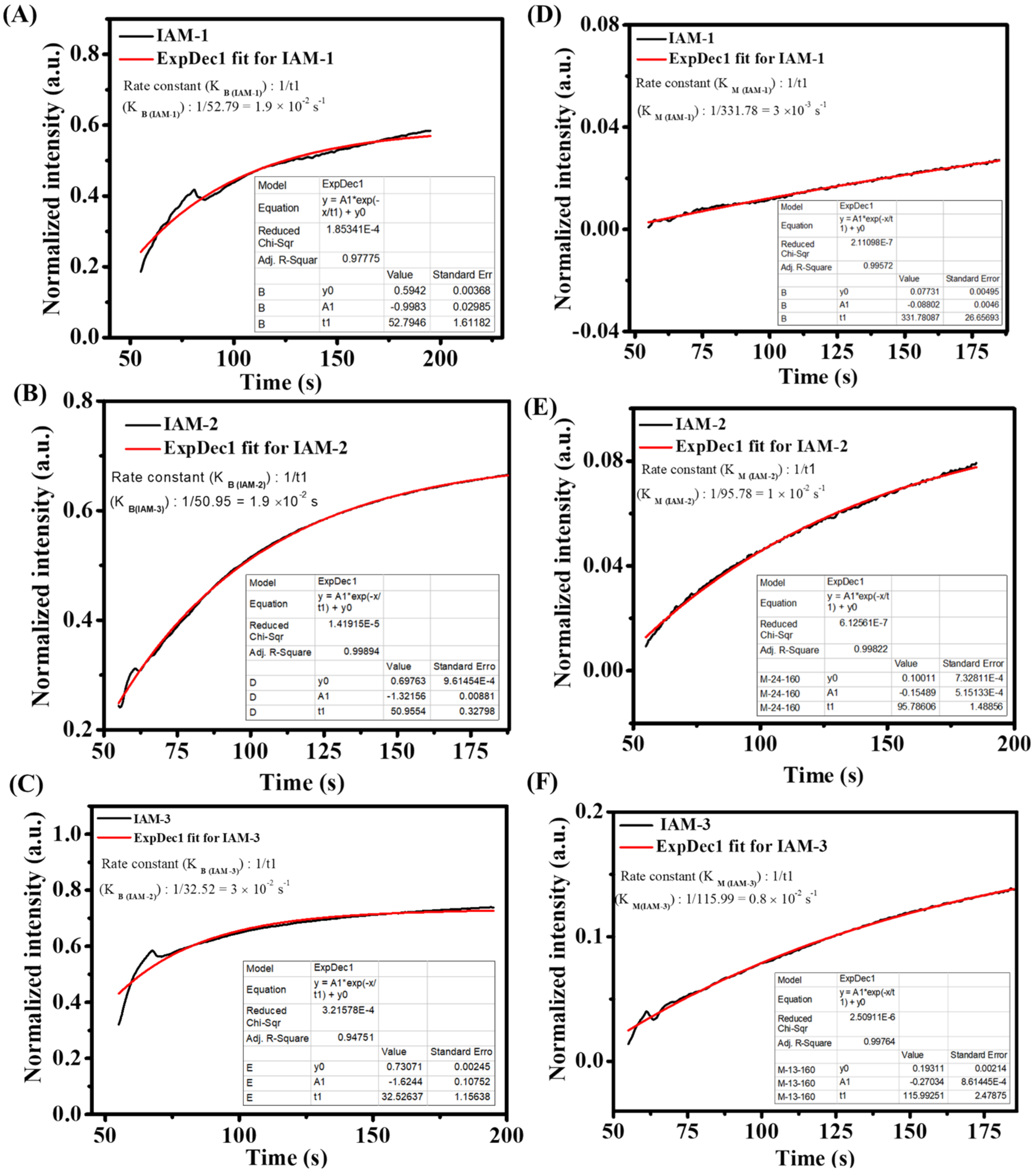

Figure S10. Representative fitting of normalized fluorescence intensity versus time plot for dye leakage assay in case of bacterial model membrane (DDPG: DPPE : 88: 12) upon treatment with (A) IAM-**1**, (B) IAM-**2** and (C) IAM-**3**. Representative fitting of normalized fluorescence intensity versus time plot for dye leakage assay in case of mammalian model membrane (DPPC) upon treatment with (A) IAM-**1**, (B) IAM-**2** and (C) IAM-**3**. Inset table shows fitting results.

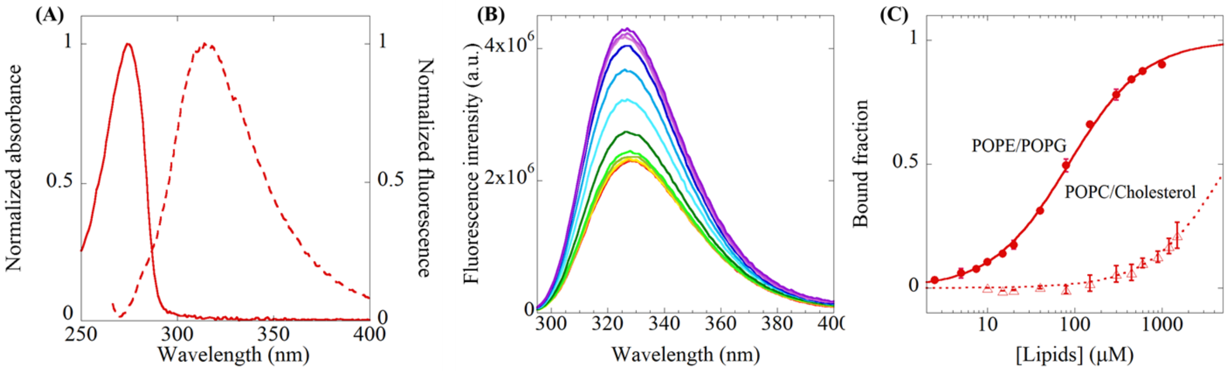

Figure S11. . (A) Normalized absorption (solid lines) and fluorescence (dashed lines) spectra of IAM-**1**. The compound concentration: 1 mM (absorbance, depending on the specific compound), 10 µM (fluorescence); water-membrane partition studies; (B) Emission spectra of IAM-**1** (10 µM) in the presence of increasing concentrations of POPE/POPG liposomes (from 0 to 1 mM, coloured from red to purple); and (C) Water-membrane partition curves with IAM-**1** [10 µM]. Filled dots and solid lines correspond to POPE/POPG 7:3 liposomes, while empty triangles and dashed lines correspond to POPC/Cholesterol 1:1 liposomes. Error bars indicate maximum error of duplicate measurements.

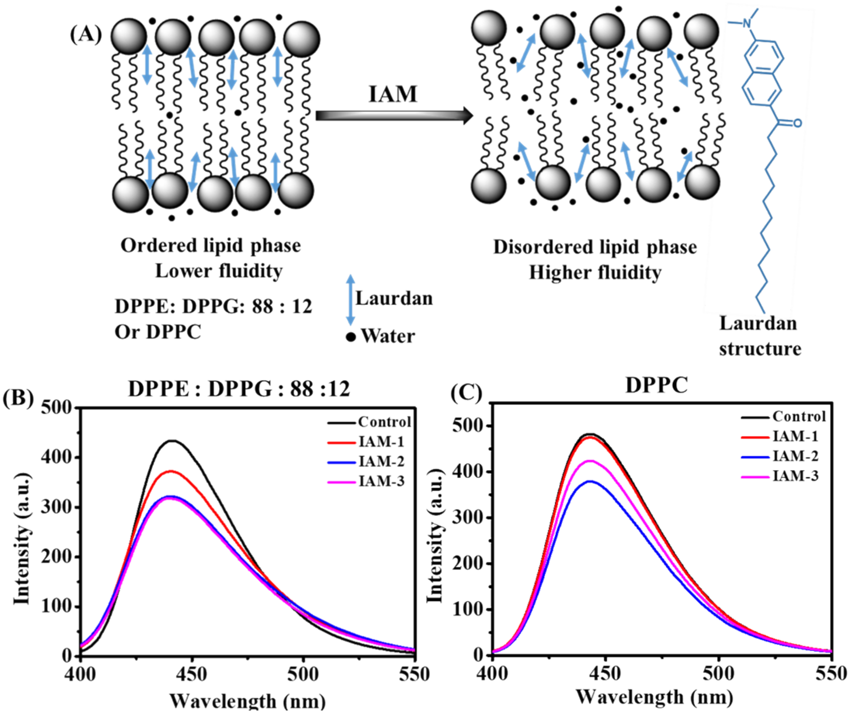

Figure S12.(A) Schematic of membrane dynamic/fluidity study using laurdan dye; (B) Change in dynamics of bacterial model membrane (DPPE: DPPG : 88: 12) upon treatment with IAMs: 1-3 at 160 µg/mL (161.1 µM) and (C) Change in dynamics of mammalian model membrane (DPPC) upon treatment with IAMs: 1-3 at 160 µg/mL (161.1 µM). Laurdan dye is well known to detect the changes in membrane fluidity. When membrane disorder allows water percolation to the region just below the head groups, where the probe is located, a spectral shift and change in intensity is observed. When the liposomes mimicking bacterial membranes were treated with IAMs **1-3** [160 µg/mL (161.1 µM)] (ortho-, meta- and para-isomers) individually, a reduction of fluorescence intensity was noticed in all the cases. This suggested that bacterial model membrane was slightly perturbed by all the positional isomers upon allowing the penetration of surrounding water molecules below the lipid head groups. Interestingly, ortho-isomer, IAM-**1** did not alter the emission spectrum of laurdan dye in case of mammalian model membrane, although it had a mild effect on bacterial model membrane. However, along with the perturbation of bacterial model membrane, IAM-**2** (meta-isomer) and IAM-**3** (para-isomer) upon interacting with mammalian model membrane decreased the fluorescence intensity.

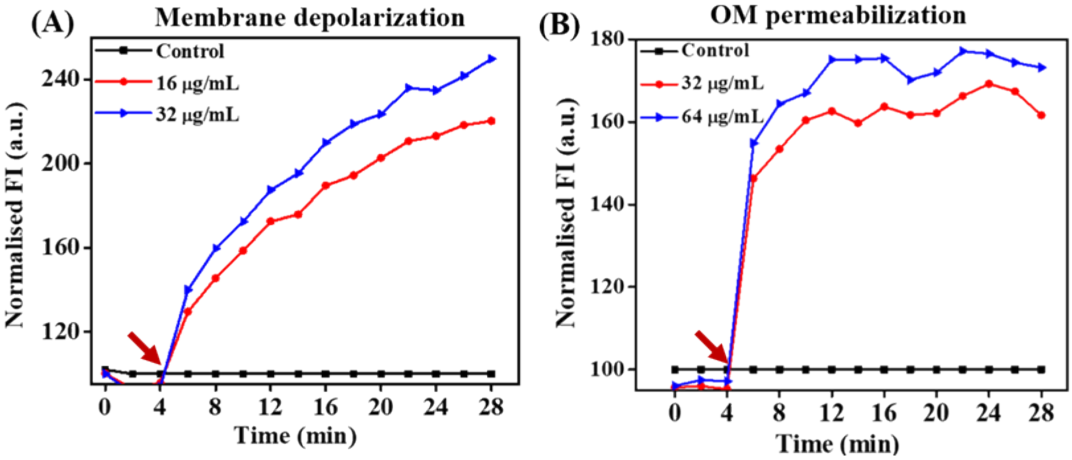

Figure S13. (A) Membrane depolarization upon IAM-**1** treatment with MRSA ATCC 33591 and (B) Outer membrane permeabilization upon IAM-**1** treatment with *E. coli* MTCC 443. The red arrow indicated the addition of IAM-**1** to the bacterial suspension.

Table 1. Antibacterial activity and hemolytic activity of isoamphipathic antibacterial molecules (IAMs: **1-3**).

Compounds	Minimum inhibitory concentration [µg/mL or (µM)]								HC_{50} (µM)
	Gram-positive bacteria						Gram-negative bacteria		
	S. aureus	MRSA	VRSA	*S. epidermidis*	*E. faecium*	VRE	*E.coli*	*A. baumannii*	
**IAM-1**	4 (4)	4 (4)	8 (8)	1 (1)	8 (8)	32 (32.2)	16 (16.1)	64 (64.4)	650 (654.6)
**IAM-2**	1 (1)	2 (2)	2 (2)	1 (1)	2 (2)	8 (8)	4 (4)	16 (16.1)	98 (98.7)
**IAM-3**	1 (1)	4 (4)	2 (2)	1 (1)	4 (4)	8 (8)	4 (4)	16 (16.1)	160 (161.1)
**Vancomycin**	1 (1)	1 (1)	1 (1)	1 (1)	1 (1)	256 (257.8)	N.D.	N.D	N.D.
**Colistin**	N.D.	N.D.	N.D.	N.D.	N.D.	N.D.	1 (1)	1 (1)	N.D.

Minimum inhibitory concentrations mentioned within the parenthesis are in micromolar (µM) unit.

References

1. C. Ghosh, G. B. Manjunath, P. Akkapeddi, V. Yarlagadda, J. Hoque, D. S. S. Uppu, M. M. Konai, J. Haldar, *J. Med. Chem.*, 2014, **57**, 1428–1436.
2. S. Barman, G. Dhanda, P. Naik, R. Mukherjee, L. Jolly, J. Joseph, J. Haldar, *Adv. Ther.*, 2022, doi.org/10.1002/adtp.202100234.
3. S. Barman, M. M. Konai, S. Samaddar, J. Haldar, *ACS. Appl. Mater. Interfaces*, 2019, **11**, 33559–33572.
4. F. Savini, V. Luca, A. Bocedi, R. Massoud, Y. Park, M. L. Mangoni, L. Stella, *ACS Chem. Biol.*, 2016, **12**, 52–56.

5. M. M. Konai, S. Samaddar, G. Bocchinfuso, V. Santucci, L. Stella, J. Haldar, *Chem. Commun.* 2018, **54**, 4943–4946.
6. D. S. S. M. Uppu, M. M. Konai, U. Baul, P. Singh, T. K. Siersma, S. Samaddar, S. Vemparala, L. W. Hamoen, C. Narayana, J. Haldar, *Chem. Sci.*, 2016, **7**, 4613–4623.
7. K. G. Varnava, A. S. Mohid, P. Calligari, L. Stella, J. Reynisson, A. Bhunia, V. Sarojini, *Bioconjug. Chem.*, 2019, **30**, 1998-2010.
8. J. C. M. Stewart, *Anal. Biochem.*, 1980, **104**, 10-14.
9. L. Stella, C. Mazzuca, M. Venanzi, A. Palleschi, M. Didonè, F. Formaggio, C. Toniolo, Pispisa *BiophyS. J.*,2004, **86**, 936-945.
10. A. K. Malde, L. Zuo, M. Breeze, M. Stroet, D. Poger, P. C. Nair, C. Oostenbrink, A. E. Mark, A. E. *J Chem Theory Comput.*, 2011, ***7***, 4026-4037.
11. S. Nathan, P. Andreas, C. Alexandra *et al. Eur. Biophys. J.*, 2011, **40**, 843-856.
12. E. Lindahl, M. J. Abraham, B. Hess, D. van der Spoel, *GROMACS 2020 Manual.* https://doi.org/10.5281/zenodo.3562512
13. A. Farrotti, G. Bocchinfuso, A. Palleschi, N. Rosato, E. S. Salnikov, N. Voievoda, B. Bechinger, L. Stella, *Biochim. Biophys. Acta.*, 2015, **1848**, 581-592.
14. B. Orioni, G. Bocchinfuso, J. Y. Kim, A. Palleschi, G. Grande, S. Bobone, K. S. Hahm, L. Stella, *Bioch. Biophys. Acta*, 2009, **1788**, 1523-1533.
15. G. Bocchinfuso, A. Palleschi, B. Orioni, G. Grande, F. Formaggio, C. Toniolo, L. Stella, *J. Pept. Sci.,*2009, **15**, 550-558.
16. O. Berger, O. Edholm, F. Jähnig, *Biophys. j*, 1997, **72**, 2002-2013.
17. G. Bussi, D. Donadio, M. Parrinello, Chem. Phys., 2007, **126**, 014101.
18. M. Parrinello, A. Rahman, *J. Appl. Phys.*, 1981, **52**, 7182-7190.
19. B. Hess, H. Bekker, H. J. Berendsen, J. G. Fraaije, *J. Comput. Chem.*, 1997, **18**, 1463-1472.
20. U. Essmann, L. Perera, M. L. Berkowitz, T. Darden, H. Lee, L. G. Pedersen, *Chem. Phys.*, 1995, **103**, 8577-8593.
21. E. F. Pettersen, T. D. Goddard, C. C. Huang, G. S. Couch, D. M. Greenblatt, E. C. Meng, T. E. Ferrin, *J. Comput. Chem.*, 2004, **25**, 1605-1612.
